# Long-term intermittent fasting improves neurological function by promoting angiogenesis after cerebral ischemia via growth differentiation factor 11 signaling activation

**DOI:** 10.1371/journal.pone.0282338

**Published:** 2023-03-30

**Authors:** Zhao Liu, Mengjie Liu, Gongwei Jia, Jiani Li, Lingchuan Niu, Huiji Zhang, Yunwen Qi, Houchao Sun, Liang-Jun Yan, Jingxi Ma

**Affiliations:** 1 Department of Neurology, Chongqing General Hospital, Chongqing, China; 2 Chongqing Key Laboratory of Neurodegenerative Diseases, Chongqing, China; 3 Department of Neurology, People’s Hospital of Chongqing Banan District, Chongqing, China; 4 Department of Rehabilitation, The Second Affiliated Hospital of Chongqing Medical University, Chongqing, China; 5 Department of Neurology, The Second Affiliated Hospital of Chongqing Medical University, Chongqing, China; 6 Department of Pharmaceutical Sciences, College of Pharmacy, University of North Texas Health Science Center, Fort Worth, TX, United States of America; Massachusetts General Hospital/Harvard Medical School, UNITED STATES

## Abstract

Intermittent fasting (IF), an alternative to caloric restriction, is a form of time restricted eating. IF conditioning has been suggested to have neuroprotective effects and potential long-term brain health benefits. But the mechanism underlying remains unclear. The present study focused on the cerebral angiogenesis effect of IF on ischemic rats. Using a rat middle cerebral artery occlusion model, we assessed neurological outcomes and various vascular parameters such as microvessel density (MVD), regional cerebral blood flow (rCBF), proliferation of endothelial cells (ECs), and functional vessels in the peri-infarct area. IF conditioning ameliorated the modified neurological severity score and adhesive removal test, increased MVD, and activated growth differentiation factor 11 (GDF11)/activin-like kinase 5 (ALK5) pathways in a time-dependent manner. In addition, long-term IF conditioning stimulated proliferation of ECs, promoted rCBF, and upregulated the total vessel surface area as well as the number of microvessel branch points through GDF11/ALK5 pathways. These data suggest that long-term IF conditioning improves neurological outcomes after cerebral ischemia, and that this positive effect is mediated partly by angiogenesis in the peri-infarct area and improvement of functional perfusion microvessels in part by activating the GDF11/ALK5 signaling pathway.

## Introduction

Intermittent fasting (IF) is a dietary intervention for various meal timing schedules that cycle between fasting and free access to food over a given period [1]. IF can prolong the healthy lifetime of the brain by regulating fundamental metabolic and cellular signaling pathways. Hence, it is a potential diet for successful brain aging [2]. A growing body of evidence has suggested that IF has neuroprotective effects through the activation of multiple signaling pathways [3], which could promote a sustained improvement in human health [4]. But its underlying mechanism is not completely clear.

Growth differentiation factor 11 (GDF11), a circulating factor, may participate in or mediate rejuvenating effects (such as remodeling of neurovascular units and enhancing neurogenesis) in old mice [5, 6]. These findings suggest that both IF and GDF11 may be potential brain aging interventions, which appear to operate through similar rejuvenating mechanisms. However, the relationship between the two has never been investigated.

Ischemic stroke is one of the leading causes of disability and death among the elderly worldwide. As peri-infarct cerebral cortex is dependent on the collateral circulation from newly sprouted vessels for blood supply, augmentation of angiogenesis could be of therapeutic value for ischemic stroke [7]. Previous studies have demonstrated that IF is protective in ischemic stroke [8]. It may reduce ischemic tissue injury and neurological deficits by inhibition of excitotoxicity, oxidative stress, neuroinflammation, or apoptosis pathways in ischemic animal models. Further exploration of the precise mechanisms of IF will help to a better understanding of the benefits of this potential therapeutic intervention.

Our previous research [9] has suggested that GDF11 is an angiogenic factor following cerebral ischemia (CI). Circulating GDF11 effectively improves neurobehavioral recovery and promotes proliferation of endothelial cells (ECs), vascular surface area, and the number of vascular branch points. However, these proangiogenic effects are suppressed by blocking activin-like kinase 5 (ALK5), the GDF11 receptor. These novel findings prompted us to further investigate whether IF could stimulate cerebral angiogenic responses after acute ischemic stroke through the GDF11/ALK5 pathway.

To test the above hypothesis, we used a rat CI model to determine whether 1) IF conditioning activated GDF11/ALK5 signals under ischemia condition; 2) IF conditioning stimulated cerebral angiogenesis; and 3) GDF11 upregulation induced by IF was the chief factor contributing to the protective effects following CI.

## Materials and methods

### Animals, diets and grouping

Adult male Sprague Dawley rats weighing 220–240 g were purchased from the Experimental Animal Center of Chongqing Medical University and housed under a 12/12 h dark/light cycle and specific pathogen-free and controlled conditions (relative humidity of 55% and room temperature of 22°C). All experimental procedures were performed in strict accordance with the guidelines of the China Animal Protection Law and were approved by Ethics Committee of Chongqing General Hospital (No. 2020022).

The diet regimen in this study used the *ad libitum* (AL) or IF diet protocol. The IF diet followed a previous study [10] with some modifications. Rats were fed within strict time periods for 8 h out of every 24 h, with free access to food between 0800 and 1600 h. Water was freely available during the IF period.

Our experiment had two steps. The first step included five groups of rats, while the second step included three groups (Table 1). Rats in the IF groups were kept on the IF diet during the recovery period after middle cerebral artery occlusion (MCAO). We monitored whether IF accelerated neurobehavioral recovery and induced the expression of ECs after MCAO in the first step. Then we explored whether GDF11 and downstream signals mediated angiogenesis in the peri-infarct area in the second step.

**Table 1:** Animal grouping for 2 steps in this study.

	Diet regimen	Diet period	MCAO	SB431542	Group abbreviation
Step 1					
1	AL	3 months			Control
2	AL	3 months	√		CI + AL
3	IF	10 days	√		CI + IF_10d_
4	IF	1 month	√		CI + IF_1m_
5	IF	3 months	√		CI + IF_3m_
Step 2					
1	AL	3 months	√		AL
2	IF	3 months	√		IF
3	IF	3 months	√	√	IF + SB

Abbreviations: AL, *ad libitum;* CI, cerebral ischemia; IF, intermittent fasting; MCAO, middle cerebral artery occlusion; SB, SB431542.

A total of 218 rats were included in the study. Data are reported on 80 rats (step 1) and 96 rats (step 2). Forty-two animals were excluded from the study because they didn’t meet the reduced rCBF criterion or died. A researcher blinded to this study performed the randomization of animals using a random number table.

### Rat MCAO model

The model was established in enflurane-anesthetized rats as described previously [11, 12]. Briefly, a nylon filament suture with rounded tip [13] (diameter = 0.26–0.28 mm), was inserted carefully from the right external carotid artery into the internal carotid artery and advanced until encountering mild resistance to blocked blood flow at the branch point of the middle cerebral artery (MCA). At the same time, changes in regional cerebral blood flow (rCBF) were continuously monitored by using a laser Doppler probe (see below in section measurement of rCBF). The body temperature was maintained at 37°C throughout the experiment. It was considered as a successful CI model when rCBF reduction in the MCA region reached at least 75% of the baseline.

In the control group, sham surgery was performed using the same anesthesia and incision in the neck. The external carotid artery was isolated, but the filament was not inserted.

### ALK5 inhibitor administration

SB431542 (MedChem Express, Monmouth Junction, NJ, USA) is an effective small molecular inhibitor that can selectively inhibit ALK5. This inhibitor was dissolved in 10% ethanol at a concentration of 0.5 mg/mL. Rats in the IF + SB group were treated with intraperitoneal injection at a dose of 4.2 mg/kg daily after CI until they were sacrificed according to a previous study [9].

### Modified neurological severity score test

Neurological function was evaluated employing the modified neurological severity score (mNSS) test, which encompassed a series of movement, sensation, reflex, and balance [14]. The neurological function score was graded on a scale ranging from 0 to 18 (0, normal score; 18, maximal deficit score). The higher the mNSS score, the more severe the neurobehavioral deficit. The performance of each rat was recorded 3 times, and the mean value was taken for analysis. The neurological function assessment was performed by a researcher blinded to the experimental groups.

### Adhesive removal somatosensory test

Sensorimotor deficits were assessed as described in a previous study [15]. We utilized two circular paper patches (diameter: 10 mm) as bilateral tactile stimuli occupying the distal-radial area on the wrist in of each rat forelimb. The mean duration of adhesive patches removal in three experiments was calculated (maximum time limit: 120 s). We separated individual tests at least 30 min to minimize individual errors. All rats were familiarized with the environment and pre-trained for a week before CI until each rat could remove the adhesive patches within 15 s.

### rCBF measurement

The rCBF in the peri-infarct area was measured using a laser Doppler (PeriFlux System 5000; Perimed, Stockholm, Sweden) as previously described [16], with the flowmeter probe placed over the right frontoparietal cortical area supplied by the MCA. Recording was performed through the exposed skull under anesthesia. The majority of the periosteum, which adheres to the skull, was carefully removed. Changes in rCBF were recorded just before MCAO (baseline), as well as 24 h and 7, 14 d after MCAO. To minimize variability, rCBF was measured for 3 min each time. The relative rCBF data after MCAO were expressed as a percentage of the baseline values.

### Enzyme-linked immunosorbent assay (ELISA)

Blood samples were extracted from the abdominal aorta 3 d after CI. The concentrations of GDF11 protein in plasma were measured using commercial ELISA kits (Colorful Gene Biological Technology, Wuhan, China), according to manufacturer’s instructions. A microplate reader (Bio Tek Instruments, Inc., Winooski, VT, USA) at a wavelength of 450 nm was used to assess the optical density value. The results were calculated according to the manufacturer’s formula.

### Immunohistochemistry

Animals were perfused transcardially with 0.9% saline at 4°C followed by 4% paraformaldehyde in phosphate buffer (pH 7.4) under anesthesia as previously described [9]. Rats were sacrificed and brains were quickly removed and fixed. Thereafter, paraffin sections (4-μm thick) were prepared for both immunohistochemistry and immunofluorescence. The expression levels of CD34 and GDF11 proteins in the peri-infarct area were detected by immunohistochemistry. Briefly, sections were deparaffinized in xylene and rehydrated in graded ethanol, and antigen was then retrieved for 20 min at 98°C. After blocking with goat serum for 20 min at 37°C, brain sections were incubated with rabbit anti-rat CD34 antibody (1:100; Abcam, Cambridge, UK) and rabbit anti-rat GDF11 antibody (1:100; Abcam, Cambridge, UK) at 4°C overnight. Then, the sections were incubated with goat anti-rabbit second antibody for 30 min at 37°C. Positive activity was revealed with diaminobenzidine coloration. For negative controls, the primary antibodies were replaced by PBS. Ten non-continuous visual fields (400×) in the peri-infarct area were randomly selected for each sample under the microscope (Olympus DP70, Tokyo, Japan). CD34 staining was regarded as an indicator of microvessel density (MVD). Any cells stained brown in the cytoplasm were considered as countable capillaries [17]. We counted the number of CD34 positive cells in each field to assess the MVD for analysis.

### Immunofluorescence analysis

Double immunofluorescence staining was carried out to visualize the cellular colocalization of CD31 and Ki67 for proliferating ECs 7 d after MCAO. After deparaffinization, dehydration, and antigen recovery, the sections were incubated with the following primary antibodies: anti-CD31 (1:100; Abcam, Cambridge, UK), and anti-Ki67 (1:100; Santa Cruz, Dallas, TX, USA) antibodies overnight at 4°C. The sections were then rinsed in PBS and incubated with secondary antibodies for 1 h at 37°C. The nuclei were labeled away in the dark with DAPI (Beyotime C1005, Shanghai, China) for 2 min. Finally, images of sections were captured using a laser scanning confocal microscope (Leica TCS SP8, Wetzlar, Germany). Ten visual fields were selected randomly from a ×40 objective lens in 290.63 μm × 290.63 μm format in the x-y direction.

### Western blotting

Rats were anesthetized and decapitated on a given day, and brain tissues from the ipsilateral peri-infarct area were collected and extracted. Anti-ALK5 (1:1000, Abcam), anti-Smad2/3 (1:1000; Santa Cruz) and anti-pSmad2/3 (1:1000; Santa Cruz) were used as primary antibodies. After the secondary antibody reaction, the bands were visualized by electrochemiluminescence (Millipore, Darmstadt, Germany). The positive pixel area was detected by an image analysis software (BIO-RAD Gel Doc 2000, Watertown, MA, USA). Western blot quantification was performed by densitometry and was normalized to GAPDH. All experiments were conducted under the same conditions and repeated three times.

### Functional vessels analysis

We labeled and analyzed functional vessels as in a previous study [18]. Fluorescein isothiocyanate (FITC)-dextran (2 × 10^6^ molecular weight, Sigma-Aldrich; 1 ml of 50 mg/ml) was injected intravenously into anesthetized rats 14 d after MCAO. Rat brains were rapidly harvested after FITC-dextran circulating adequately in cerebral intact perfused microvessels for 2 min. Coronal brain thick sections (100-μm) were then cut on a vibratome after fixed in 4% paraformaldehyde at 4°C for 48 h. Eight fields in the peri-infarct area were randomly selected for analysis. An investigator blinded to the group calculated the vascular surface area (mm^2^) with the Image-Pro Plus software (Media Cybernetics, Inc., Bethesda, MD, USA) and counted the number of vascular branch points under a microscope (Olympus).

### Statistical analysis

Statistical analyses were performed using SPSS software version 21. All data were presented as the mean ± standard deviation. One-way ANOVA followed by the least significant difference tests was used for multiple-group comparisons. A value of p < 0.05 was considered to represent statistically significant differences.

## Results

### Long-term IF conditioning promotes neurological function recovery 7 d after CI

**Fig 1:**
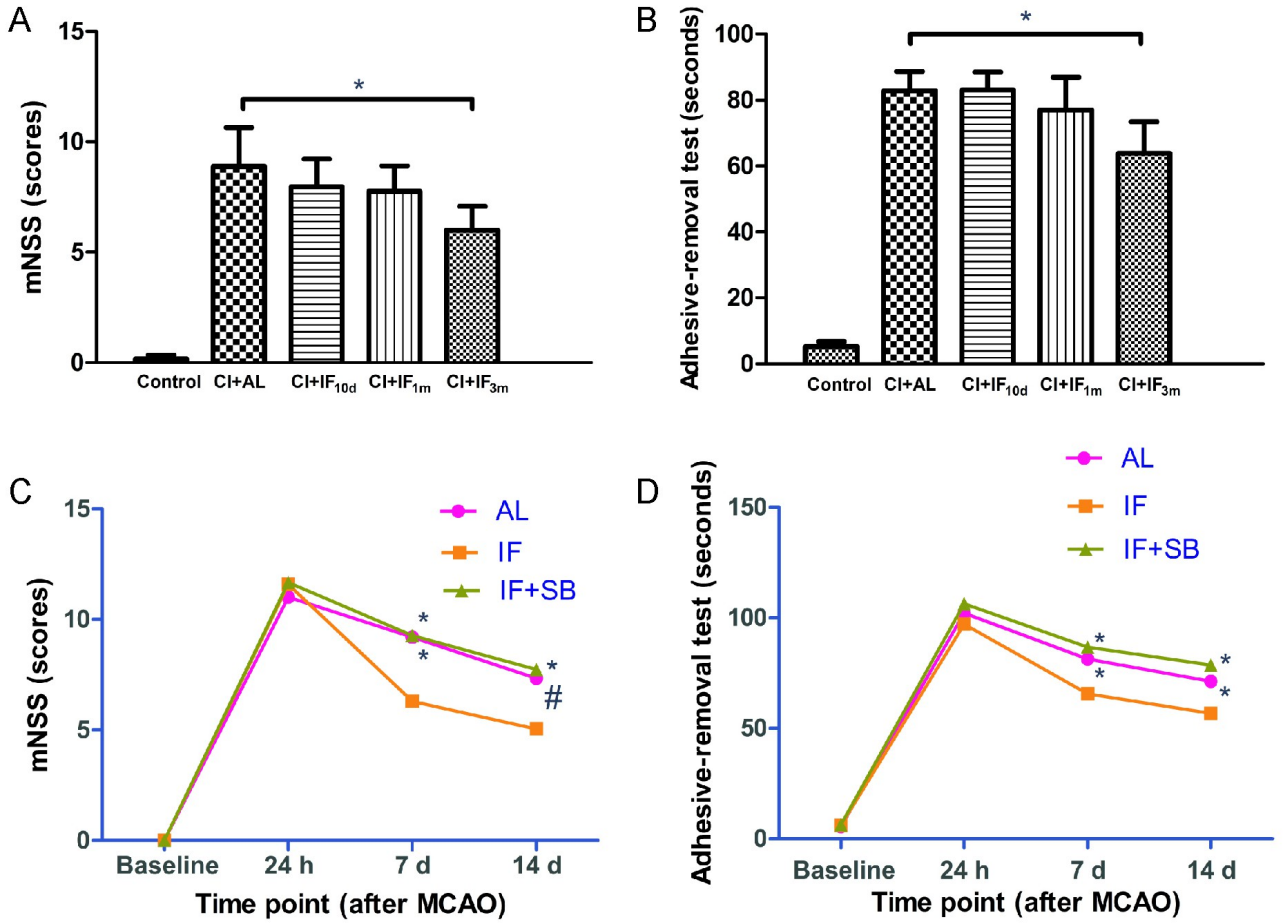
Neurological function assessment results in each group. mNSS (**A**) and adhesive removal test (**B**) results in different dietary regimens of the 5 animal groups from the first step of this study 7 d after CI (n = 8/group). * p < 0.01, compared with the CI+AL group. mNSS (**C**) and adhesive removal test (**D**) results in different time points of 3 groups from the second step of this study (n = 16/group). # p < 0.05, * p < 0.01, compared with the IF group at the same time point.

In the first step, we designed different time schedules (10 d, 1 month, and 3 months) of IF before MCAO in rats. Time-dependent improvements in scores of both mNSS and adhesive removal test were discovered 7 d after CI (Fig 1A and 1B). However, only results in the CI + IF_3m_ group were statistically different compared with those of the CI + AL group (p < 0.01). These findings suggested that the longer the period of IF before MCAO, the better the protective effects after surgery.

### Long-term IF conditioning increases MVD in the peri-infarct area 7 d after CI

**Fig 2:**
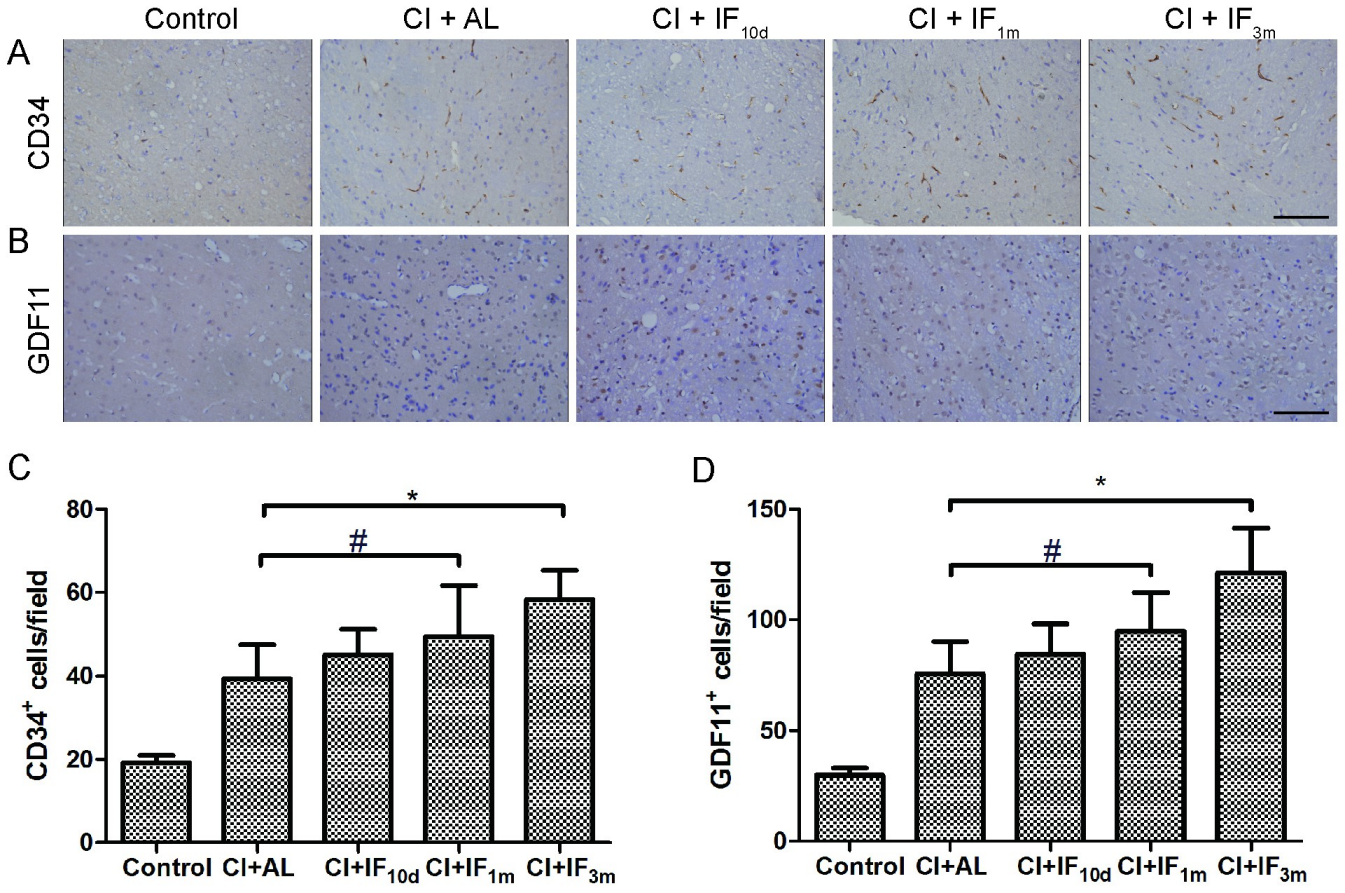
(**A**) Long-term IF conditioning promotes the expression level of cerebral CD34 7 d after MCAO. Scale bar, 100 μm (**B**) Long-term IF conditioning promotes the expression level of cerebral GDF11 protein 3 d after MCAO. Scale bar, 100 μm Representative images of immunohistochemistry for CD34 and GDF11 in the peri-infarct area are shown (original magnification ×400; scale bar, 100 μm). Bar graphs showing the CD31^+^ (**C**) and GDF11^+^ (**D**) cells/mm^2^ in the 5 groups. # p < 0.05, * p < 0.01 compared with the CI + AL group (n = 8/group).

To test whether IF pretreatment induces a proangiogenic effect, the expression of CD34 was detected by immunohistochemistry (Fig 2A). Overall, there was a time-dependent increase trend in MVD induced by IF in the peri-infarct area. Expression of CD34 in the peri-infarct area was much higher 7 d after MCAO in the CI + IF_1m_ (p < 0.05) and CI + IF_3m_ (p < 0.01) groups compared with the CI + AL group. IF for only 10 d before surgery promoted MVD in the peri-infarct area 7 d after CI, with no significant differences observed between the CI + AL group and the CI + IF_10d_ group (p > 0.05) (Fig 2C). Thus, our novel findings showed that preoperative long-term IF might be beneficial for inducing cerebral angiogenesis in acute cerebral infarction.

### Long-term IF conditioning activates GDF11/ALK5/Smad2/3 pathways after CI

We found that 3 months (p < 0.01) and 1 month (p < 0.05) of IF conditioning, respectively, markedly increased GDF11-positive cells in the peri-infarct area 3 d after MCAO as observed by immunohistochemistry (Fig 2B) compared with AL dietary regimen. However, there were no significant differences between the CI + AL group and the CI + IF_10d_ group (p > 0.05) (Fig 2D).

**Fig 3:**
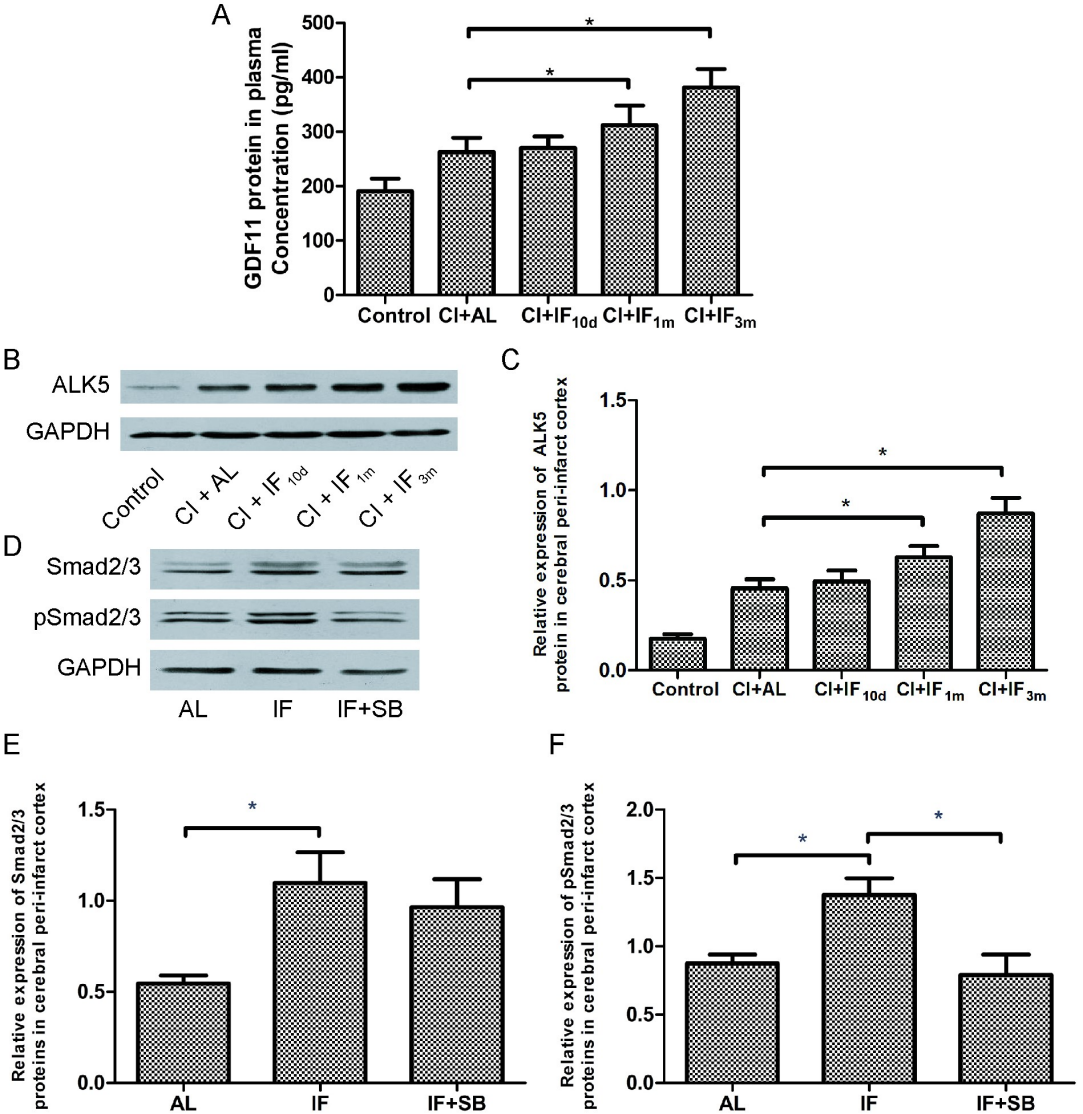
Long-term IF conditioning activates GDF11/ALK5/ Smad2/3 pathways after CI. (**A**) IF upregulates the expression level of circulating GDF11 3 d after CI. Bar graph showing GDF11 protein in plasma in the 5 groups as indicated by ELISA. * p < 0.01 compared with the CI + AL group (n = 8 /group). (**B**) IF upregulates the expression level of cerebral ALK5 3 d after CI. Representative images of western blot showing ALK5 expression in the peri-infarct area from the first step of this study. (**C**) Bar graph showing ALK5 protein expression level (relative to GAPDH) in the 5 groups. * p < 0.01 compared with the CI + AL group (n = 8/group). (**D**) Representative images of western blot showing total Smad2/3 and pSmad2/3 expression in the peri-infarct area 7 d after CI from the second step of this study. Bar graph showing Smad2/3 (**E**) and pSmad2/3 (**F**) protein expression levels (relative to GAPDH) in the 3 groups. * p < 0.01 compared with the IF group (n = 8/group).

GDF11 was regarded as a circulating factor; therefore, we also assayed the plasma expression pattern of GDF11 protein. ELISA showed that the plasma level of GDF11 protein was significantly upregulated in the IF dietary groups compared with the AL dietary group (CI + IF_1m_, p < 0.01; CI + IF_3m_ p < 0.01) 3 d after MCAO, which was consistent with the brain level. However, short-term (10 d) IF conditioning results were not statistically different. (CI + AL group vs. CI + IF_10d_ group, p > 0.05) (Fig 3A). Taken together, our results strongly indicated that pretreatment of long-term IF might promote circulation of GDF11 and cerebral GDF11 protein during the post-ischemic, recovery period.

Western blotting showed that the expression of ALK5 protein was significantly upregulated in the peri-infarct cerebral cortex of the CI + IF_1m_ group (p < 0.01) and the CI + IF_3m_ group (p < 0.01) with respect to the CI + AL group 3 d after MCAO (Fig 3B and 3C). However, IF conditioning for 10 d did not have this promoting effect (p > 0.05). These observations demonstrated that pre-intervention with long-term IF activates GDF11 downstream signal ALK5. Moreover, this also means that longer IF enhances activation of the cerebral ALK5 signal (CI + IF_1m_ group vs. CI + IF_3m_ group, p < 0.01). Based on the preceding results, we used a 3-month IF dietary regimen in the second step of this study.

In the second step, we detected total Smad2/3 (Fig 3D and 3E) and phosphorylated Smad2/3 (pSmad2/3) (Fig 3D and 3F) in the peri-infarct area using western blotting 7 d after MCAO. Consistent with the result obtained from the GDF11/ALK5 data, total Smad2/3 and pSmad2/3 protein levels significantly increased with long-term IF conditioning (IF group vs. AL group, p < 0.01). In general, long-term IF thus activates GDF11/ALK5/Smad2/3 pathways after CI. However, the pSmad2/3 protein level was markedly reduced in the IF + SB group compared with the IF group (p < 0.01), which suggested that SB431542, an ALK5 inhibitor, blocked phosphorylation of Smad2/3.

### Long-term IF conditioning promotes neurological function recovery at different time points after CI through the GDF11/ALK5 pathways

In this section, we found that long-term IF conditioning accelerated neurofunctional recovery 7 d and 14 d after MCAO, consistent with our findings from the first step. However, the ameliorating effect of IF was inhibited by SB431542. mNSS scores and the seconds of the adhesive removal somatosensory test in the IF + SB group were significantly decreased in comparison with the IF group in 7 d (p < 0.01) and 14 d (p < 0.01) after MCAO (Fig 1C and 1D). These data strongly suggest that long-term IF conditioning promotes functional neurological recovery after CI through the GDF11/ALK5 pathways.

### Long-term IF conditioning stimulates proliferation of ECs after CI through the GDF11/ALK5 pathways

**Fig 4:**
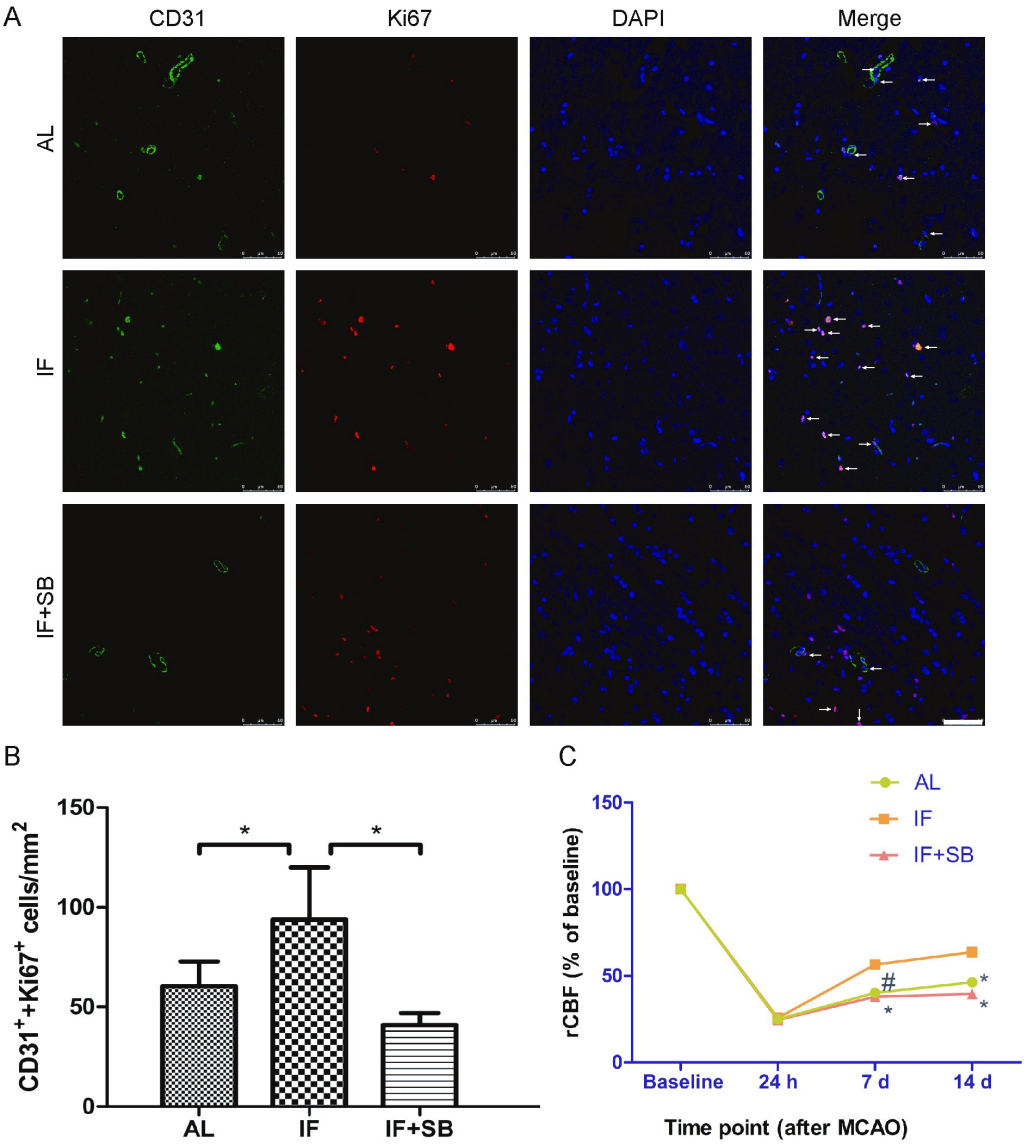
Long-term IF conditioning stimulates proliferation of ECs and improves rCBF after CI through the GDF11/ALK5 pathways. (**A**) Proliferation of ECs 7 d after MCAO in each group as indicated by immunofluorescence. Arrows show the colocalization of CD31 (green stain) and Ki67 (red stain) in each group. Scale bar, 50 μm. (**B**) Bar graph showing the CD31^+^/Ki67^+^ cells/mm^2^ in each group. * p < 0.01 in comparison with the IF group (n = 8/group). (**C**) rCBF (relative to baseline) as indicated by laser Doppler flowmetry in each group. # p < 0.05, * p < 0.01 compared with the IF group at the same time point (n = 8/group).

We also found that 1 week after CI, rats in the IF group had significantly increased co-expression of CD31 and ki67 in comparison with AL controls (p < 0.01). Moreover, this increased expression was blocked by inhibition of ALK5 (IF group vs. IF + SB group, p < 0.01) (Fig 4A and 4B). These findings indicate that long-term IF conditioning stimulates proliferation of ECs after CI and likely occurs through the GDF11/ALK5 pathways.

### Long-term IF conditioning improves rCBF after CI through the GDF11/ALK5 pathways

Laser Doppler flowmetry was used to investigate rCBF in the MCA regions after CI. As shown in Fig 4C and IF rats displayed significant increases in rCBF compared with AL rats (7 d, p < 0.05; 14 d, p < 0.01). However, rats from the IF + SB group showed significant decreases in the rCBF compared to those from the IF group 7 d and 14 d after CI (p < 0.01). These results indicate that long-term IF conditioning may promote poststroke rCBF.

### Long-term IF conditioning promotes both vascular surface area and the number of vascular branch points after CI through the GDF11/ALK5 pathways

**Fig 5:**
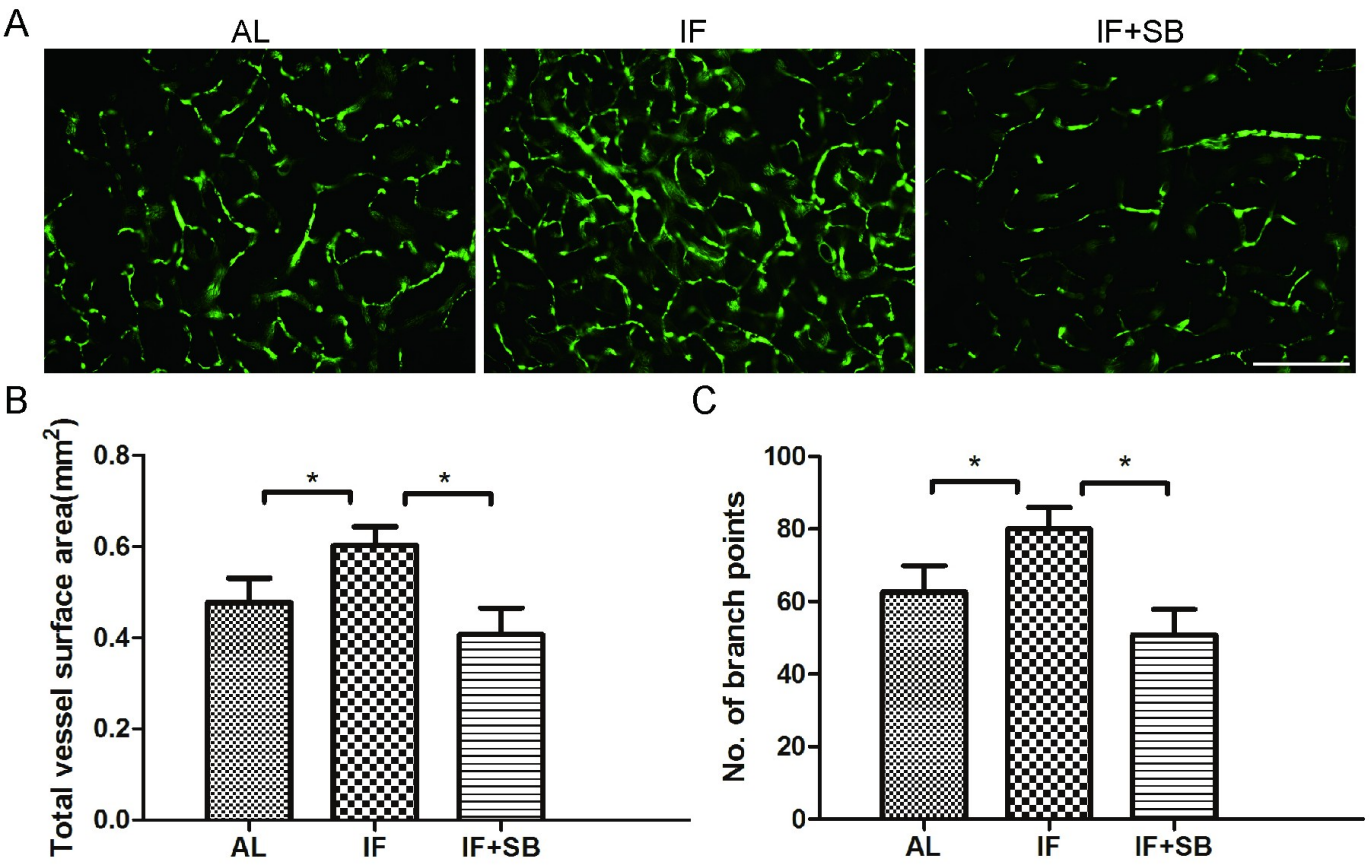
Long-term IF conditioning promotes functional perfusion of microvessels in the peri-infarct area 14 d after CI. (**A**) Cerebral functional microvessels labeled with FITC-dextran in each group. Scale bar, 200 μm. Bar graphs showing total vessel surface area (**B**) and the number of vessel branch points (**C**) in each group. * p < 0.01 (n = 8/group).

Functional vessels were labeled with perfused FITC-dextran. Results showed that long-term IF conditioning remarkably increased the total vessel surface area (p < 0.01) and the number of vascular branch points (p < 0.01) in the peri-infarct area at 14 d after CI, compared with the AL control group. We further found that SB431542 inhibited these enhancing effects in total vessel surface area (IF group vs. IF + SB group, p < 0.01) and the number of vascular branch points (IF group vs. IF + SB group, p < 0.01), (Fig 5A–5C). In general, long-term IF conditioning promotes functional perfusion of microvessels 14 d after MCAO.

## Discussion

Reduction of calorie intake throughout life (caloric restriction, CR) significantly affects animal aging and lifespan [19]. CR, in the absence of malnutrition, has so far been considered a non-genetic intervention that has consistently been found to prolong both mean and maximum lifespans in various species [20].

While straightforward, in real human life, consistently reducing calorie or food intake doesn’t seem to be easy and truly achievable. In recent years, IF has emerged as an alternative of CR that is likely to trigger similar biological pathways [21].

Regular IF is a strict dietary regimen that induces energy restriction through planned alternating periods of fasting and AL [8]. IF conditioning, which has many potentially physical and psychologic benefits, has been shown to prolong lifespan and reduce the progression and severity of age-related vascular diseases. Relative to CR, IF is not only more patient-friendly and sustainable, but also prevents some adverse effects of chronic CR. Studies of controlled IF regimens demonstrate metabolic conversion from hepatocyte-derived glucose to adipocyte–derived ketone bodies occurs daily or several days per week [19].

IF has recently gained popularity for its multiple potential long-term health benefits, such as inhibiting progression of atherosclerosis and regulating blood sugar, blood pressure, and other vascular risk factors. Growing evidence has suggested that IF plays a protective role in various neurological disorders, such as Parkinson’s disease [22], multiple sclerosis [22], Alzheimer’s disease [23], epilepsy [23], and ischemic stroke [8], as well as other diseases. Recent research shows that preventive IF may reduce ischemic injury and neurological deficits in animal stroke models [8]. IF may be an effective treatment for acute cerebral ischemic injury and for neuroprotection.

Recent study has shown that prolonged fasting promotes endothelial progenitor cell-mediated cerebral angiogenesis and improves long-term outcomes of acute stroke in mice, which may mean that prolonged fasting may be a new therapeutic strategy for ischemic stroke [24]. However, IF is a scheduled periodic energy restriction that has been shown to have health benefits nearly equivalent to CR or extended fasting [25]. A 6-month randomized pilot study [26] suggested that intermittent and continuous energy restriction resulted in similar health effects, such as weight loss and changes in bodily composition. Obviously, intermittent intervention has better adherence and sustainability. IF is a safe, well-controlled, economical, effective, repeatable, non-pharmaceutical intervention measure. IF may act as a mild metabolic stressor for neurons or glial cells, leading to upregulation of neurotrophic factors, stress response proteins, regulatory proteins, antioxidant enzymes, and uncoupling proteins [8], which potentially could help accelerate the recovery from pathological injury that ocurrs during acute ischemic stroke.

Our present study demonstrated that long-term IF conditioning promotes poststroke neurological function recovery and improves various vascular parameters, such as MVD, rCBF, proliferation of ECs, vascular surface area, and number of vascular branch points in the peri-infarct area. Moreover, this preventive measure has a gradual, cumulative effect. By extending this intervention, the protective effect may be enhanced, which suggests that a person’s long-term adherence to an IF diet regimen will enable stronger recovery and improved vascular regeneration in ischemic stroke.

Growing evidences suggested that exogenous systemic interventions such as CR, young blood and circulating GDF11 may partially counteract age-related progressive loss of neuroplasticity in the aged brain [27–29]. However, whether these two systemic interventions, CR and GDF11, are directly or indirectly related has scarcely been researched until recently. In this study [30], circulating GDF11 induces CR-like phenotypic effects, increases adiponectin secretion, and promotes cerebral neurogenesis in aged mice. These results suggest that GDF11 is a pleiotropic factor at the organismal level, which may serve as a bridging mechanism for rejuvenating effects between heterochronic parabiosis and CR.

**Fig 6:**
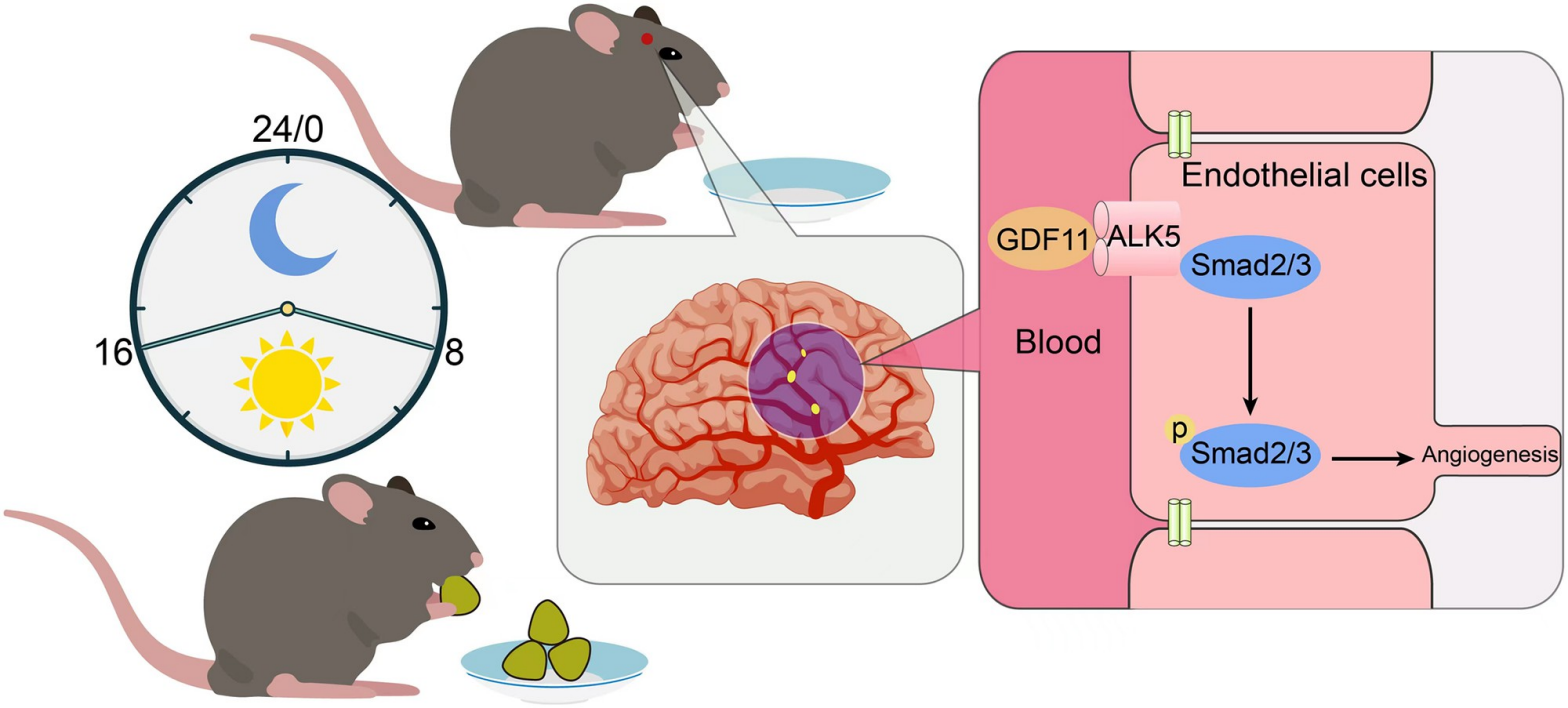
Schematic diagram of IF on cerebral angiogenesis hypothesis. Long-term IF promotes metabolism and energy conversion, and activates GDF11/ALK5 signaling pathway in blood circulation. Accompanied by phosphorylation of downstream signaling Smad2/3, cerebral angiogenesis is further activated. The promotion of neurological recovery induced by IF in ischemic stroke is partly mediated by its angiogenic effects.

The aging process of brain means a gradual decline in memory and cognitive function, accompanied by a slow loss of neurons and neurological deficits. The potential of GDF11 as a “rejuvenating factor” [31] opens up prospects for the treatment of age-related neurodegenerative disease. As a promising neurogenic and angiogenic factor, GDF11 may form the basis of potential therapeutic strategy for acute ischemic stroke. In a cohort study, GDF11 played a key role in various metabolic processes and was closely associated with metabolic syndrome in the Chinese population [32]. The present research indicates a specific relationship between IF and GDF11 signaling in the context of CI (Fig 6). Long-term IF conditioning activates GDF11/ALK5/Smad2/3 pathways in rats subjected to CI, which suggests that GDF11 signals mediate the protective effects of IF. The proangiogenic effects of long-term IF conditioning disappeared when ALK5 was blocked. Our study confirmed that both circulating and cerebral GDF11 protein were elevated after CI. The underlying mechanism by which GDF11 in the blood circulation induced by IF acts on the central nervous system needs to be further explored.

In summary, our novel findings provide a unique perspective on the potential mechanism of IF, a non-pharmacological intervention that induces angiogenic effects associated with CI. Our results suggest that long-term IF conditioning improves recovery from neurological deficits after CI injury and that this positive effect is partly mediated by proangiogenesis and expansion of the functional perfusion of microvessels, possibly through GDF11/ALK5 pathways.

## Supporting information

S1 File (DOCX)

S2 File (DOCX)

S1 Raw image (JPG)
