# Supplementary material for: Long-term intermittent fasting improves neurological function by promoting angiogenesis after cerebral ischemia via growth differentiation factor 11 signaling activation
Source: PLoS One. 2023 Mar 30;18(3):e0282338. doi: 10.1371/journal.pone.0282338 (PMC10062670; doi:10.1371/journal.pone.0282338)
Supplement: S1 File — (DOCX) [file pone.0282338.s001.docx]

|  |  |  |  |  |
| --- | --- | --- | --- | --- |
| mNSS | | | | |
| Control group (n=8) | | | | |
| Control 1 | 0 | 0 | 0 |  |
| Control 2 | 0 | 0 | 1 |  |
| Control 3 | 0 | 1 | 0 |  |
| Control 4 | 0 | 0 | 0 |  |
| Control 5 | 0 | 0 | 0 |  |
| Control 6 | 1 | 0 | 0 |  |
| Control 7 | 0 | 1 | 0 |  |
| Control 8 | 0 | 0 | 0 |  |
| CI+AL group (n=8) | | | | |
| CI+AL 1 | 10 | 11 | 12 |  |
| CI+AL 2 | 8 | 8 | 9 |  |
| CI+AL 3 | 14 | 11 | 10 |  |
| CI+AL 4 | 8 | 7 | 6 |  |
| CI+AL 5 | 9 | 7 | 6 |  |
| CI+AL 6 | 9 | 10 | 11 |  |
| CI+AL 7 | 7 | 8 | 8 |  |
| CI+AL 8 | 8 | 9 | 7 |  |
| CI+IF_10d_ group (n=8) | | | | |
| CI+IF_10d_ 1 | 8 | 8 | 8 |  |
| CI+IF_10d_ 2 | 7 | 7 | 5 |  |
| CI+IF_10d_ 3 | 11 | 9 | 11 |  |
| CI+IF_10d_ 4 | 8 | 8 | 8 |  |
| CI+IF_10d_ 5 | 6 | 6 | 8 |  |
| CI+IF_10d_ 6 | 9 | 10 | 8 |  |
| CI+IF_10d_ 7 | 8 | 6 | 9 |  |
| CI+IF_10d_ 8 | 8 | 8 | 7 |  |
| CI+IF_1m_ group (n=8) | | | | |
| CI+IF_1m_ 1 | 9 | 8 | 8 |  |
| CI+IF_1m_ 2 | 10 | 9 | 8 |  |
| CI+IF_1m_ 3 | 7 | 5 | 6 |  |
| CI+IF_1m_ 4 | 9 | 9 | 7 |  |
| CI+IF_1m_ 5 | 8 | 10 | 8 |  |
| CI+IF_1m_ 6 | 6 | 8 | 9 |  |
| CI+IF_1m_ 7 | 8 | 8 | 8 |  |
| CI+IF_1m_ 8 | 7 | 7 | 4 |  |
| CI+IF_3m_ group (n=8) | | | | |
| CI+IF_3m_ 1 | 7 | 8 | 8 |  |
| CI+IF_3m_ 2 | 5 | 6 | 4 |  |
| CI+IF_3m_ 3 | 4 | 5 | 4 |  |
| CI+IF_3m_ 4 | 6 | 6 | 5 |  |
| CI+IF_3m_ 5 | 7 | 5 | 6 |  |
| CI+IF_3m_ 6 | 7 | 7 | 6 |  |
| CI+IF_3m_ 7 | 5 | 4 | 8 |  |
| CI+IF_3m_ 8 | 6 | 7 | 8 |  |
|  |  |  |  |  |

|  |  |  |  |  |
| --- | --- | --- | --- | --- |
| Adhesive-removal test | | | | |
| Control group (n=8) | | | | |
| Control 1 | 4 | 3 | 5 |  |
| Control 2 | 3 | 2 | 6 |  |
| Control 3 | 4 | 3 | 6 |  |
| Control 4 | 6 | 4 | 7 |  |
| Control 5 | 4 | 5 | 4 |  |
| Control 6 | 6 | 6 | 3 |  |
| Control 7 | 6 | 6 | 8 |  |
| Control 8 | 8 | 8 | 9 |  |
| CI+AL group (n=8) | | | | |
| CI+AL 1 | 90 | 65 | 87 |  |
| CI+AL 2 | 80 | 85 | 96 |  |
| CI+AL 3 | 70 | 86 | 76 |  |
| CI+AL 4 | 79 | 78 | 88 |  |
| CI+AL 5 | 81 | 90 | 96 |  |
| CI+AL 6 | 88 | 86 | 92 |  |
| CI+AL 7 | 85 | 82 | 91 |  |
| CI+AL 8 | 87 | 70 | 61 |  |
| CI+IF_10d_ group (n=8) | | | | |
| CI+IF_10d_ 1 | 98 | 87 | 86 |  |
| CI+IF_10d_ 2 | 87 | 87 | 88 |  |
| CI+IF_10d_ 3 | 82 | 109 | 67 |  |
| CI+IF_10d_ 4 | 83 | 80 | 84 |  |
| CI+IF_10d_ 5 | 56 | 97 | 102 |  |
| CI+IF_10d_ 6 | 86 | 45 | 87 |  |
| CI+IF_10d_ 7 | 95 | 69 | 79 |  |
| CI+IF_10d_ 8 | 75 | 76 | 90 |  |
| CI+IF_1m_ group (n=8) | | | | |
| CI+IF_1m_ 1 | 68 | 55 | 85 |  |
| CI+IF_1m_ 2 | 115 | 78 | 84 |  |
| CI+IF_1m_ 3 | 79 | 87 | 57 |  |
| CI+IF_1m_ 4 | 100 | 81 | 68 |  |
| CI+IF_1m_ 5 | 99 | 68 | 94 |  |
| CI+IF_1m_ 6 | 66 | 76 | 84 |  |
| CI+IF_1m_ 7 | 46 | 98 | 76 |  |
| CI+IF_1m_ 8 | 79 | 58 | 47 |  |
| CI+IF_3m_ group (n=8) | | | | |
| CI+IF_3m_ 1 | 68 | 78 | 60 |  |
| CI+IF_3m_ 2 | 87 | 75 | 82 |  |
| CI+IF_3m_ 3 | 54 | 66 | 60 |  |
| CI+IF_3m_ 4 | 62 | 89 | 55 |  |
| CI+IF_3m_ 5 | 83 | 72 | 39 |  |
| CI+IF_3m_ 6 | 51 | 63 | 70 |  |
| CI+IF_3m_ 7 | 68 | 47 | 58 |  |
| CI+IF_3m_ 8 | 55 | 44 | 48 |  |
|  |  |  |  |  |

|  |  |  |  |  |  |  |  |  |  |  |
| --- | --- | --- | --- | --- | --- | --- | --- | --- | --- | --- |
| CD34 Immunohistochemistry （cells/Field） | | | | | | | | | | |
| Control group (n=8) | | | | | | | | | | |
| Control 1 | 28 | 25 | 14 | 11 | 30 | 17 | 18 | 22 | 17 | 24 |
| Control 2 | 22 | 20 | 31 | 12 | 14 | 18 | 25 | 10 | 16 | 19 |
| Control 3 | 21 | 16 | 26 | 25 | 16 | 16 | 19 | 14 | 23 | 25 |
| Control 4 | 12 | 16 | 15 | 17 | 10 | 23 | 21 | 16 | 24 | 20 |
| Control 5 | 14 | 19 | 21 | 25 | 21 | 16 | 24 | 15 | 16 | 21 |
| Control 6 | 25 | 26 | 21 | 31 | 16 | 17 | 13 | 30 | 23 | 21 |
| Control 7 | 13 | 13 | 18 | 26 | 8 | 15 | 21 | 18 | 16 | 18 |
| Control 8 | 16 | 17 | 12 | 25 | 27 | 21 | 15 | 12 | 25 | 12 |
| CI+AL group (n=8) | | | | | | | | | | |
| CI+AL 1 | 48 | 51 | 63 | 45 | 42 | 39 | 44 | 47 | 32 | 35 |
| CI+AL 2 | 34 | 27 | 29 | 35 | 37 | 43 | 55 | 42 | 26 | 40 |
| CI+AL 3 | 25 | 40 | 46 | 34 | 52 | 36 | 37 | 31 | 25 | 30 |
| CI+AL 4 | 45 | 66 | 34 | 37 | 55 | 52 | 58 | 37 | 35 | 68 |
| CI+AL 5 | 26 | 35 | 43 | 26 | 15 | 28 | 15 | 32 | 13 | 31 |
| CI+AL 6 | 39 | 35 | 32 | 31 | 38 | 36 | 35 | 31 | 29 | 45 |
| CI+AL 7 | 53 | 61 | 38 | 44 | 76 | 51 | 42 | 47 | 53 | 45 |
| CI+AL 8 | 31 | 26 | 57 | 36 | 32 | 48 | 31 | 36 | 39 | 26 |
| CI+IF_10d_ group (n=8) | | | | | | | | | | |
| CI+IF_10d_ 1 | 57 | 68 | 45 | 47 | 35 | 44 | 51 | 35 | 45 | 27 |
| CI+IF_10d_ 2 | 46 | 35 | 47 | 55 | 53 | 36 | 38 | 40 | 51 | 55 |
| CI+IF_10d_ 3 | 35 | 46 | 19 | 52 | 49 | 24 | 39 | 42 | 26 | 35 |
| CI+IF_10d_ 4 | 33 | 56 | 55 | 48 | 51 | 62 | 66 | 35 | 46 | 37 |
| CI+IF_10d_ 5 | 25 | 45 | 55 | 56 | 27 | 46 | 21 | 43 | 29 | 37 |
| CI+IF_10d_ 6 | 51 | 47 | 25 | 32 | 22 | 43 | 47 | 52 | 36 | 47 |
| CI+IF_10d_ 7 | 46 | 65 | 49 | 45 | 63 | 42 | 68 | 37 | 44 | 60 |
| CI+IF_10d_ 8 | 66 | 61 | 42 | 47 | 62 | 41 | 38 | 72 | 44 | 60 |
| CI+IF_1m_ group (n=8) | | | | | | | | | | |
| CI+IF_1m_ 1 | 66 | 78 | 57 | 76 | 60 | 86 | 63 | 53 | 53 | 46 |
| CI+IF_1m_ 2 | 46 | 35 | 39 | 34 | 52 | 34 | 26 | 32 | 45 | 48 |
| CI+IF_1m_ 3 | 62 | 83 | 69 | 72 | 77 | 59 | 48 | 58 | 62 | 51 |
| CI+IF_1m_ 4 | 47 | 18 | 36 | 33 | 42 | 49 | 37 | 25 | 46 | 37 |
| CI+IF_1m_ 5 | 36 | 14 | 47 | 22 | 29 | 46 | 16 | 38 | 57 | 44 |
| CI+IF_1m_ 6 | 30 | 53 | 23 | 54 | 48 | 37 | 47 | 61 | 40 | 33 |
| CI+IF_1m_ 7 | 47 | 69 | 46 | 86 | 43 | 68 | 47 | 50 | 42 | 86 |
| CI+IF_1m_ 8 | 48 | 67 | 58 | 54 | 48 | 58 | 54 | 48 | 59 | 61 |
| CI+IF_3m_ group (n=8) | | | | | | | | | | |
| CI+IF_3m_ 1 | 68 | 98 | 75 | 89 | 52 | 64 | 57 | 58 | 37 | 61 |
| CI+IF_3m_ 2 | 58 | 81 | 69 | 76 | 78 | 65 | 48 | 47 | 34 | 67 |
| CI+IF_3m_ 3 | 57 | 68 | 77 | 76 | 71 | 54 | 57 | 51 | 59 | 42 |
| CI+IF_3m_ 4 | 47 | 68 | 75 | 56 | 58 | 52 | 55 | 46 | 58 | 32 |
| CI+IF_3m_ 5 | 52 | 36 | 67 | 47 | 55 | 64 | 66 | 36 | 42 | 37 |
| CI+IF_3m_ 6 | 35 | 68 | 79 | 50 | 60 | 57 | 47 | 72 | 36 | 51 |
| CI+IF_3m_ 7 | 69 | 82 | 84 | 78 | 67 | 58 | 52 | 55 | 68 | 65 |
| CI+IF_3m_ 8 | 50 | 43 | 57 | 46 | 47 | 59 | 68 | 39 | 35 | 46 |
|  |  |  |  |  |  |  |  |  |  |  |

|  |  |  |  |  |  |  |  |  |  |  |
| --- | --- | --- | --- | --- | --- | --- | --- | --- | --- | --- |
| GDF11 Immunohistochemistry （cells/Field） | | | | | | | | | | |
| Control group (n=8) | | | | | | | | | | |
| Control 1 | 32 | 34 | 37 | 23 | 26 | 29 | 30 | 26 | 32 | 31 |
| Control 2 | 24 | 36 | 26 | 35 | 21 | 25 | 32 | 25 | 36 | 28 |
| Control 3 | 32 | 23 | 31 | 35 | 24 | 20 | 33 | 32 | 29 | 31 |
| Control 4 | 24 | 26 | 34 | 24 | 27 | 21 | 24 | 25 | 16 | 21 |
| Control 5 | 31 | 25 | 40 | 36 | 31 | 30 | 38 | 34 | 35 | 38 |
| Control 6 | 25 | 36 | 31 | 34 | 28 | 46 | 25 | 39 | 31 | 41 |
| Control 7 | 27 | 34 | 31 | 26 | 47 | 23 | 46 | 37 | 23 | 26 |
| Control 8 | 21 | 15 | 25 | 22 | 27 | 31 | 26 | 54 | 21 | 26 |
| CI+AL group (n=8) | | | | | | | | | | |
| CI+AL 1 | 98 | 104 | 123 | 118 | 87 | 103 | 90 | 76 | 127 | 100 |
| CI+AL 2 | 89 | 67 | 90 | 87 | 48 | 75 | 80 | 73 | 87 | 76 |
| CI+AL 3 | 69 | 80 | 63 | 57 | 79 | 72 | 77 | 81 | 84 | 87 |
| CI+AL 4 | 79 | 72 | 77 | 71 | 67 | 82 | 74 | 47 | 80 | 79 |
| CI+AL 5 | 65 | 80 | 75 | 73 | 72 | 99 | 82 | 85 | 78 | 39 |
| CI+AL 6 | 69 | 78 | 93 | 79 | 101 | 82 | 93 | 107 | 89 | 75 |
| CI+AL 7 | 67 | 28 | 47 | 81 | 36 | 78 | 40 | 69 | 64 | 78 |
| CI+AL 8 | 79 | 41 | 36 | 37 | 86 | 75 | 76 | 43 | 47 | 52 |
| CI+IF_10d_ group (n=8) | | | | | | | | | | |
| CI+IF_10d_ 1 | 101 | 98 | 87 | 74 | 89 | 107 | 79 | 79 | 88 | 84 |
| CI+IF_10d_ 2 | 87 | 94 | 78 | 68 | 39 | 92 | 87 | 68 | 88 | 81 |
| CI+IF_10d_ 3 | 67 | 90 | 58 | 52 | 53 | 84 | 82 | 89 | 81 | 83 |
| CI+IF_10d_ 4 | 79 | 60 | 81 | 58 | 53 | 59 | 70 | 62 | 66 | 64 |
| CI+IF_10d_ 5 | 98 | 100 | 118 | 88 | 96 | 91 | 94 | 108 | 92 | 95 |
| CI+IF_10d_ 6 | 118 | 108 | 103 | 98 | 93 | 88 | 82 | 87 | 90 | 97 |
| CI+IF_10d_ 7 | 124 | 107 | 94 | 101 | 105 | 112 | 92 | 87 | 75 | 126 |
| CI+IF_10d_ 8 | 47 | 75 | 68 | 89 | 90 | 80 | 88 | 67 | 76 | 46 |
| CI+IF_1m_ group (n=8) | | | | | | | | | | |
| CI+IF_1m_ 1 | 132 | 140 | 150 | 108 | 114 | 112 | 110 | 109 | 135 | 128 |
| CI+IF_1m_ 2 | 79 | 73 | 80 | 82 | 95 | 77 | 106 | 73 | 87 | 90 |
| CI+IF_1m_ 3 | 50 | 123 | 116 | 126 | 114 | 109 | 123 | 118 | 109 | 100 |
| CI+IF_1m_ 4 | 125 | 118 | 116 | 121 | 86 | 88 | 101 | 100 | 124 | 103 |
| CI+IF_1m_ 5 | 68 | 80 | 52 | 57 | 80 | 76 | 78 | 74 | 72 | 71 |
| CI+IF_1m_ 6 | 89 | 83 | 87 | 99 | 76 | 108 | 109 | 95 | 98 | 90 |
| CI+IF_1m_ 7 | 79 | 83 | 82 | 89 | 88 | 92 | 76 | 78 | 74 | 93 |
| CI+IF_1m_ 8 | 88 | 86 | 80 | 83 | 98 | 92 | 93 | 78 | 72 | 102 |
| CI+IF_3m_ group (n=8) | | | | | | | | | | |
| CI+IF_3m_ 1 | 99 | 86 | 83 | 85 | 132 | 87 | 73 | 77 | 80 | 81 |
| CI+IF_3m_ 2 | 145 | 125 | 150 | 136 | 142 | 123 | 128 | 120 | 126 | 129 |
| CI+IF_3m_ 3 | 101 | 108 | 103 | 115 | 114 | 134 | 130 | 132 | 135 | 131 |
| CI+IF_3m_ 4 | 145 | 138 | 137 | 138 | 146 | 124 | 156 | 119 | 150 | 133 |
| CI+IF_3m_ 5 | 132 | 135 | 145 | 149 | 150 | 132 | 122 | 127 | 127 | 117 |
| CI+IF_3m_ 6 | 88 | 91 | 99 | 102 | 84 | 113 | 79 | 86 | 88 | 83 |
| CI+IF_3m_ 7 | 145 | 136 | 132 | 135 | 138 | 139 | 142 | 133 | 126 | 129 |
| CI+IF_3m_ 8 | 130 | 116 | 98 | 143 | 147 | 144 | 134 | 125 | 136 | 129 |
|  |  |  |  |  |  |  |  |  |  |  |

|  |  |  |  |  |
| --- | --- | --- | --- | --- |
| GDF11 ELISA | | | | |
| Control group (n=8) | | | | |
| Control 1 | 0.12625 | 0.15367 | 0.18285 |  |
| Control 2 | 0.15873 | 0.17276 | 0.13782 |  |
| Control 3 | 0.17267 | 0.16234 | 0.14274 |  |
| Control 4 | 0.11873 | 0.09798 | 0.08796 |  |
| Control 5 | 0.10982 | 0.09876 | 0.08973 |  |
| Control 6 | 0.12374 | 0.08287 | 0.09283 |  |
| Control 7 | 0.13546 | 0.16135 | 0.14468 |  |
| Control 8 | 0.16576 | 0.09876 | 0.07869 |  |
| CI+AL group (n=8) | | | | |
| CI+AL 1 | 0.20001 | 0.19786 | 0.15636 |  |
| CI+AL 2 | 0.22418 | 0.25317 | 0.26543 |  |
| CI+AL 3 | 0.22154 | 0.18979 | 0.19876 |  |
| CI+AL 4 | 0.18769 | 0.1987 | 0.16879 |  |
| CI+AL 5 | 0.27687 | 0.29873 | 0.22438 |  |
| CI+AL 6 | 0.18793 | 0.1987 | 0.20984 |  |
| CI+AL 7 | 0.16574 | 0.19821 | 0.21532 |  |
| CI+AL 8 | 0.22143 | 0.20976 | 0.25431 |  |
| CI+IF_10d_ group (n=8) | | | | |
| CI+IF_10d_ 1 | 0.21342 | 0.18768 | 0.1982 |  |
| CI+IF_10d_ 2 | 0.25647 | 0.23653 | 0.26667 |  |
| CI+IF_10d_ 3 | 0.1876 | 0.26856 | 0.24327 |  |
| CI+IF_10d_ 4 | 0.22987 | 0.25678 | 0.21548 |  |
| CI+IF_10d_ 5 | 0.29831 | 0.25431 | 0.20887 |  |
| CI+IF_10d_ 6 | 0.20198 | 0.21876 | 0.22312 |  |
| CI+IF_10d_ 7 | 0.19876 | 0.18769 | 0.19721 |  |
| CI+IF_10d_ 8 | 0.18723 | 0.19876 | 0.2001 |  |
| CI+IF_1m_ group (n=8) | | | | |
| CI+IF_1m_ 1 | 0.25768 | 0.29876 | 0.22981 |  |
| CI+IF_1m_ 2 | 0.25231 | 0.24211 | 0.22101 |  |
| CI+IF_1m_ 3 | 0.2562 | 0.22139 | 0.24231 |  |
| CI+IF_1m_ 4 | 0.32176 | 0.32658 | 0.34887 |  |
| CI+IF_1m_ 5 | 0.25472 | 0.26524 | 0.2332 |  |
| CI+IF_1m_ 6 | 0.19221 | 0.26539 | 0.19879 |  |
| CI+IF_1m_ 7 | 0.34983 | 0.28589 | 0.32541 |  |
| CI+IF_1m_ 8 | 0.25325 | 0.32876 | 0.329 |  |
| CI+IF_3m_ group (n=8) | | | | |
| CI+IF_3m_ 1 | 0.40983 | 0.34987 | 0.39981 |  |
| CI+IF_3m_ 2 | 0.32452 | 0.34654 | 0.29891 |  |
| CI+IF_3m_ 3 | 0.34243 | 0.33432 | 0.32879 |  |
| CI+IF_3m_ 4 | 0.30798 | 0.29878 | 0.26781 |  |
| CI+IF_3m_ 5 | 0.35438 | 0.29876 | 0.33876 |  |
| CI+IF_3m_ 6 | 0.35431 | 0.39657 | 0.33346 |  |
| CI+IF_3m_ 7 | 0.46548 | 0.42165 | 0.35412 |  |
| CI+IF_3m_ 8 | 0.35467 | 0.36542 | 0.40543 |  |
|  |  |  |  |  |

|  |  |  |  |  |
| --- | --- | --- | --- | --- |
| ALK5 Western blot | | | | |
| Control group (n=8) | | | | |
|  | ALK5 | GAPDH |  |  |
| Control 1 | 23159.78 | 144056.8 |  |  |
| Control 2 | 24765.99 | 140983.7 |  |  |
| Control 3 | 26883.23 | 145820.1 |  |  |
| Control 4 | 32332.15 | 150991.3 |  |  |
| Control 5 | 27643.37 | 145663.4 |  |  |
| Control 6 | 26374.32 | 142987.9 |  |  |
| Control 7 | 17989.76 | 148244.2 |  |  |
| Control 8 | 23443.32 | 146983 |  |  |
| CI+AL group (n=8) | | | | |
| CI+AL 1 | 65429.41 | 143920.9 |  |  |
| CI+AL 2 | 68682.92 | 148337.8 |  |  |
| CI+AL 3 | 70265.53 | 146537.7 |  |  |
| CI+AL 4 | 63621.3 | 140367.2 |  |  |
| CI+AL 5 | 51739.21 | 138975.4 |  |  |
| CI+AL 6 | 64918.92 | 143892.1 |  |  |
| CI+AL 7 | 79837.82 | 145565.2 |  |  |
| CI+AL 8 | 62822.46 | 147815.3 |  |  |
| CI+IF_10d_ group (n=8) | | | | |
| CI+IF_10d_ 1 | 71564.12 | 148877.8 |  |  |
| CI+IF_10d_ 2 | 76838.66 | 149321.2 |  |  |
| CI+IF_10d_ 3 | 86389.34 | 142873.6 |  |  |
| CI+IF_10d_ 4 | 74637.62 | 140763.3 |  |  |
| CI+IF_10d_ 5 | 70982.43 | 144159.5 |  |  |
| CI+IF_10d_ 6 | 67647.42 | 146872.7 |  |  |
| CI+IF_10d_ 7 | 56873.11 | 143918 |  |  |
| CI+IF_10d_ 8 | 69874.02 | 147653.1 |  |  |
| CI+IF_1m_ group (n=8) | | | | |
| CI+IF_1m_ 1 | 95501.43 | 148291.7 |  |  |
| CI+IF_1m_ 2 | 100281.27 | 140981.3 |  |  |
| CI+IF_1m_ 3 | 91242.12 | 142937.4 |  |  |
| CI+IF_1m_ 4 | 89383.22 | 143872.8 |  |  |
| CI+IF_1m_ 5 | 73783.46 | 145277.4 |  |  |
| CI+IF_1m_ 6 | 85744.92 | 141983.5 |  |  |
| CI+IF_1m_ 7 | 104843.91 | 150932.8 |  |  |
| CI+IF_1m_ 8 | 90188.35 | 148376.2 |  |  |
| CI+IF_3m_ group (n=8) | | | | |
| CI+IF_3m_ 1 | 126683.8 | 140320.5 |  |  |
| CI+IF_3m_ 2 | 135432.93 | 142887.2 |  |  |
| CI+IF_3m_ 3 | 121966.7 | 147112.3 |  |  |
| CI+IF_3m_ 4 | 135771.62 | 146227.9 |  |  |
| CI+IF_3m_ 5 | 103652.53 | 149246.8 |  |  |
| CI+IF_3m_ 6 | 118636.86 | 147837.4 |  |  |
| CI+IF_3m_ 7 | 128932.67 | 140989.9 |  |  |
| CI+IF_3m_ 8 | 139283.27 | 147893.4 |  |  |
|  |  |  |  |  |
