# Supplementary material for: Long-term intermittent fasting improves neurological function by promoting angiogenesis after cerebral ischemia via growth differentiation factor 11 signaling activation
Source: PLoS One. 2023 Mar 30;18(3):e0282338. doi: 10.1371/journal.pone.0282338 (PMC10062670; doi:10.1371/journal.pone.0282338)
Supplement: S2 File — (DOCX) [file pone.0282338.s002.docx]

|  |  |  |  |  |
| --- | --- | --- | --- | --- |
| mNSS | | | | |
| AL group | | | | |
| Baseline (n=16) | | | | |
| Baseline 1 | 0 | 0 | 0 |  |
| Baseline 2 | 0 | 0 | 0 |  |
| Baseline 3 | 0 | 0 | 0 |  |
| Baseline 4 | 0 | 0 | 0 |  |
| Baseline 5 | 0 | 0 | 0 |  |
| Baseline 6 | 0 | 0 | 0 |  |
| Baseline 7 | 0 | 0 | 0 |  |
| Baseline 8 | 0 | 0 | 0 |  |
| Baseline 9 | 0 | 0 | 0 |  |
| Baseline 10 | 0 | 0 | 0 |  |
| Baseline 11 | 0 | 0 | 0 |  |
| Baseline 12 | 0 | 0 | 0 |  |
| Baseline 13 | 0 | 0 | 0 |  |
| Baseline 14 | 0 | 0 | 0 |  |
| Baseline 15 | 0 | 0 | 0 |  |
| Baseline 16 | 0 | 0 | 0 |  |
| 24 h (n=16) | | | | |
| 24 h 1 | 14 | 14 | 15 |  |
| 24 h 2 | 14 | 13 | 13 |  |
| 24 h 3 | 13 | 13 | 13 |  |
| 24 h 4 | 11 | 10 | 8 |  |
| 24 h 5 | 8 | 6 | 9 |  |
| 24 h 6 | 8 | 8 | 8 |  |
| 24 h 7 | 13 | 12 | 12 |  |
| 24 h 8 | 15 | 15 | 16 |  |
| 24 h 9 | 14 | 14 | 14 |  |
| 24 h 10 | 10 | 12 | 9 |  |
| 24 h 11 | 10 | 9 | 10 |  |
| 24 h 12 | 12 | 12 | 10 |  |
| 24 h 13 | 8 | 9 | 10 |  |
| 24 h 14 | 8 | 6 | 9 |  |
| 24 h 15 | 9 | 11 | 8 |  |
| 24 h 16 | 10 | 12 | 11 |  |
| 7 d (n=16) | | | | |
| 7 d 1 | 12 | 10 | 11 |  |
| 7 d 2 | 9 | 9 | 8 |  |
| 7 d 3 | 13 | 13 | 12 |  |
| 7 d 4 | 11 | 7 | 8 |  |
| 7 d 5 | 5 | 6 | 4 |  |
| 7 d 6 | 8 | 6 | 8 |  |
| 7 d 7 | 10 | 9 | 12 |  |
| 7 d 8 | 15 | 13 | 14 |  |
| 7 d 9 | 12 | 12 | 12 |  |
| 7 d 10 | 10 | 9 | 9 |  |
| 7 d 11 | 8 | 9 | 8 |  |
| 7 d 12 | 7 | 8 | 7 |  |
| 7 d 13 | 8 | 9 | 10 |  |
| 7 d 14 | 5 | 6 | 5 |  |
| 7 d 15 | 9 | 9 | 8 |  |
| 7 d 16 | 10 | 9 | 9 |  |
| 14 d (n=16) | | | | |
| 14 d 1 | 9 | 8 | 8 |  |
| 14 d 2 | 6 | 6 | 6 |  |
| 14 d 3 | 10 | 10 | 11 |  |
| 14 d 4 | 8 | 7 | 7 |  |
| 14 d 5 | 4 | 4 | 4 |  |
| 14 d 6 | 6 | 4 | 6 |  |
| 14 d 7 | 8 | 7 | 9 |  |
| 14 d 8 | 12 | 12 | 12 |  |
| 14 d 9 | 12 | 13 | 12 |  |
| 14 d 10 | 8 | 7 | 7 |  |
| 14 d 11 | 7 | 7 | 7 |  |
| 14 d 12 | 5 | 5 | 5 |  |
| 14 d 13 | 6 | 7 | 7 |  |
| 14 d 14 | 4 | 4 | 5 |  |
| 14 d 15 | 7 | 6 | 6 |  |
| 14 d 16 | 7 | 7 | 7 |  |
| IF group | | | | |
| Baseline (n=16) | | | | |
| Baseline 1 | 0 | 0 | 0 |  |
| Baseline 2 | 0 | 0 | 0 |  |
| Baseline 3 | 0 | 0 | 0 |  |
| Baseline 4 | 0 | 0 | 0 |  |
| Baseline 5 | 0 | 0 | 0 |  |
| Baseline 6 | 0 | 0 | 0 |  |
| Baseline 7 | 0 | 0 | 0 |  |
| Baseline 8 | 0 | 0 | 0 |  |
| Baseline 9 | 0 | 0 | 0 |  |
| Baseline 10 | 0 | 0 | 0 |  |
| Baseline 11 | 0 | 0 | 0 |  |
| Baseline 12 | 0 | 0 | 0 |  |
| Baseline 13 | 0 | 0 | 0 |  |
| Baseline 14 | 0 | 0 | 0 |  |
| Baseline 15 | 0 | 0 | 0 |  |
| Baseline 16 | 0 | 0 | 0 |  |
| 24 h (n=16) | | | | |
| 24 h 1 | 16 | 14 | 12 |  |
| 24 h 2 | 14 | 15 | 15 |  |
| 24 h 3 | 13 | 13 | 12 |  |
| 24 h 4 | 6 | 5 | 7 |  |
| 24 h 5 | 11 | 10 | 12 |  |
| 24 h 6 | 9 | 8 | 7 |  |
| 24 h 7 | 9 | 7 | 10 |  |
| 24 h 8 | 10 | 11 | 14 |  |
| 24 h 9 | 17 | 16 | 16 |  |
| 24 h 10 | 10 | 10 | 10 |  |
| 24 h 11 | 12 | 15 | 14 |  |
| 24 h 12 | 11 | 11 | 12 |  |
| 24 h 13 | 15 | 13 | 16 |  |
| 24 h 14 | 14 | 13 | 15 |  |
| 24 h 15 | 10 | 7 | 9 |  |
| 24 h 16 | 11 | 9 | 10 |  |
| 7 d (n=16) | | | | |
| 7 d 1 | 10 | 14 | 12 |  |
| 7 d 2 | 4 | 5 | 5 |  |
| 7 d 3 | 8 | 7 | 8 |  |
| 7 d 4 | 6 | 5 | 7 |  |
| 7 d 5 | 5 | 7 | 5 |  |
| 7 d 6 | 5 | 5 | 3 |  |
| 7 d 7 | 3 | 3 | 5 |  |
| 7 d 8 | 9 | 8 | 8 |  |
| 7 d 9 | 8 | 10 | 10 |  |
| 7 d 10 | 6 | 5 | 6 |  |
| 7 d 11 | 5 | 6 | 5 |  |
| 7 d 12 | 4 | 4 | 2 |  |
| 7 d 13 | 8 | 7 | 8 |  |
| 7 d 14 | 5 | 5 | 4 |  |
| 7 d 15 | 5 | 6 | 7 |  |
| 7 d 16 | 6 | 7 | 6 |  |
| 14 d (n=16) | | | | |
| 14 d 1 | 9 | 12 | 11 |  |
| 14 d 2 | 3 | 4 | 4 |  |
| 14 d 3 | 7 | 6 | 7 |  |
| 14 d 4 | 5 | 5 | 6 |  |
| 14 d 5 | 4 | 6 | 4 |  |
| 14 d 6 | 4 | 4 | 3 |  |
| 14 d 7 | 2 | 2 | 4 |  |
| 14 d 8 | 8 | 7 | 7 |  |
| 14 d 9 | 8 | 8 | 8 |  |
| 14 d 10 | 5 | 4 | 5 |  |
| 14 d 11 | 4 | 5 | 4 |  |
| 14 d 12 | 1 | 2 | 1 |  |
| 14 d 13 | 5 | 6 | 7 |  |
| 14 d 14 | 4 | 4 | 4 |  |
| 14 d 15 | 4 | 4 | 5 |  |
| 14 d 16 | 3 | 5 | 2 |  |
| IF + SB group | | | | |
| Baseline (n=16) | | | | |
| Baseline 1 | 0 | 0 | 0 |  |
| Baseline 2 | 0 | 0 | 0 |  |
| Baseline 3 | 0 | 0 | 0 |  |
| Baseline 4 | 0 | 0 | 0 |  |
| Baseline 5 | 0 | 0 | 0 |  |
| Baseline 6 | 0 | 0 | 0 |  |
| Baseline 7 | 0 | 0 | 0 |  |
| Baseline 8 | 0 | 0 | 0 |  |
| Baseline 9 | 0 | 0 | 0 |  |
| Baseline 10 | 0 | 0 | 0 |  |
| Baseline 11 | 0 | 0 | 0 |  |
| Baseline 12 | 0 | 0 | 0 |  |
| Baseline 13 | 0 | 0 | 0 |  |
| Baseline 14 | 0 | 0 | 0 |  |
| Baseline 15 | 0 | 0 | 0 |  |
| Baseline 16 | 0 | 0 | 0 |  |
| 24 h (n=16) | | | | |
| 24 h 1 | 14 | 14 | 15 |  |
| 24 h 2 | 13 | 10 | 11 |  |
| 24 h 3 | 14 | 10 | 12 |  |
| 24 h 4 | 12 | 13 | 11 |  |
| 24 h 5 | 15 | 13 | 14 |  |
| 24 h 6 | 14 | 13 | 12 |  |
| 24 h 7 | 13 | 14 | 16 |  |
| 24 h 8 | 14 | 9 | 10 |  |
| 24 h 9 | 12 | 9 | 9 |  |
| 24 h 10 | 16 | 14 | 16 |  |
| 24 h 11 | 13 | 12 | 10 |  |
| 24 h 12 | 8 | 9 | 8 |  |
| 24 h 13 | 11 | 8 | 9 |  |
| 24 h 14 | 8 | 8 | 9 |  |
| 24 h 15 | 12 | 10 | 10 |  |
| 24 h 16 | 10 | 11 | 12 |  |
| 7 d (n=16) | | | | |
| 7 d 1 | 9 | 10 | 8 |  |
| 7 d 2 | 7 | 6 | 6 |  |
| 7 d 3 | 5 | 5 | 5 |  |
| 7 d 4 | 7 | 8 | 7 |  |
| 7 d 5 | 7 | 10 | 9 |  |
| 7 d 6 | 9 | 13 | 12 |  |
| 7 d 7 | 13 | 12 | 16 |  |
| 7 d 8 | 8 | 9 | 10 |  |
| 7 d 9 | 8 | 9 | 9 |  |
| 7 d 10 | 16 | 14 | 13 |  |
| 7 d 11 | 13 | 12 | 9 |  |
| 7 d 12 | 7 | 6 | 8 |  |
| 7 d 13 | 7 | 8 | 9 |  |
| 7 d 14 | 8 | 8 | 9 |  |
| 7 d 15 | 8 | 10 | 10 |  |
| 7 d 16 | 10 | 11 | 12 |  |
| 14 d (n=16) | | | | |
| 14 d 1 | 7 | 8 | 6 |  |
| 14 d 2 | 3 | 4 | 4 |  |
| 14 d 3 | 3 | 1 | 3 |  |
| 14 d 4 | 4 | 4 | 4 |  |
| 14 d 5 | 5 | 6 | 7 |  |
| 14 d 6 | 7 | 11 | 10 |  |
| 14 d 7 | 11 | 10 | 10 |  |
| 14 d 8 | 8 | 9 | 9 |  |
| 14 d 9 | 8 | 8 | 7 |  |
| 14 d 10 | 14 | 14 | 13 |  |
| 14 d 11 | 11 | 12 | 12 |  |
| 14 d 12 | 5 | 6 | 6 |  |
| 14 d 13 | 6 | 6 | 8 |  |
| 14 d 14 | 8 | 8 | 8 |  |
| 14 d 15 | 8 | 8 | 8 |  |
| 14 d 16 | 11 | 11 | 11 |  |
|  |  |  |  |  |

|  |  |  |  |  |
| --- | --- | --- | --- | --- |
| Adhesive-removal test | | | | |
| AL group | | | | |
| Baseline (n=16) | | | | |
| Baseline 1 | 6 | 7 | 4 |  |
| Baseline 2 | 5 | 4 | 2 |  |
| Baseline 3 | 4 | 4 | 2 |  |
| Baseline 4 | 5 | 7 | 6 |  |
| Baseline 5 | 6 | 7 | 8 |  |
| Baseline 6 | 7 | 3 | 8 |  |
| Baseline 7 | 10 | 5 | 1 |  |
| Baseline 8 | 2 | 5 | 9 |  |
| Baseline 9 | 4 | 6 | 9 |  |
| Baseline 10 | 6 | 8 | 8 |  |
| Baseline 11 | 10 | 4 | 5 |  |
| Baseline 12 | 10 | 3 | 4 |  |
| Baseline 13 | 4 | 2 | 3 |  |
| Baseline 14 | 5 | 8 | 2 |  |
| Baseline 15 | 6 | 6 | 6 |  |
| Baseline 16 | 2 | 9 | 4 |  |
| 24 h (n=16) | | | | |
| 24 h 1 | 120 | 120 | 120 |  |
| 24 h 2 | 98 | 120 | 98 |  |
| 24 h 3 | 78 | 109 | 97 |  |
| 24 h 4 | 90 | 100 | 80 |  |
| 24 h 5 | 115 | 87 | 99 |  |
| 24 h 6 | 120 | 120 | 115 |  |
| 24 h 7 | 120 | 118 | 115 |  |
| 24 h 8 | 110 | 120 | 98 |  |
| 24 h 9 | 98 | 116 | 97 |  |
| 24 h 10 | 98 | 65 | 87 |  |
| 24 h 11 | 116 | 88 | 100 |  |
| 24 h 12 | 120 | 118 | 120 |  |
| 24 h 13 | 108 | 105 | 88 |  |
| 24 h 14 | 88 | 70 | 67 |  |
| 24 h 15 | 98 | 105 | 88 |  |
| 24 h 16 | 89 | 98 | 105 |  |
| 7 d (n=16) | | | | |
| 7 d 1 | 101 | 118 | 88 |  |
| 7 d 2 | 90 | 84 | 87 |  |
| 7 d 3 | 68 | 85 | 96 |  |
| 7 d 4 | 70 | 66 | 70 |  |
| 7 d 5 | 79 | 76 | 88 |  |
| 7 d 6 | 87 | 67 | 61 |  |
| 7 d 7 | 98 | 78 | 89 |  |
| 7 d 8 | 76 | 87 | 100 |  |
| 7 d 9 | 67 | 68 | 87 |  |
| 7 d 10 | 84 | 75 | 97 |  |
| 7 d 11 | 28 | 78 | 65 |  |
| 7 d 12 | 89 | 98 | 74 |  |
| 7 d 13 | 78 | 82 | 56 |  |
| 7 d 14 | 74 | 89 | 109 |  |
| 7 d 15 | 108 | 120 | 100 |  |
| 7 d 16 | 78 | 54 | 36 |  |
| 14 d (n=16) | | | | |
| 14 d 1 | 60 | 68 | 79 |  |
| 14 d 2 | 80 | 85 | 68 |  |
| 14 d 3 | 87 | 97 | 81 |  |
| 14 d 4 | 75 | 67 | 60 |  |
| 14 d 5 | 82 | 89 | 81 |  |
| 14 d 6 | 58 | 58 | 59 |  |
| 14 d 7 | 97 | 108 | 92 |  |
| 14 d 8 | 86 | 83 | 60 |  |
| 14 d 9 | 76 | 82 | 55 |  |
| 14 d 10 | 34 | 47 | 52 |  |
| 14 d 11 | 81 | 65 | 43 |  |
| 14 d 12 | 65 | 64 | 30 |  |
| 14 d 13 | 78 | 63 | 76 |  |
| 14 d 14 | 37 | 65 | 79 |  |
| 14 d 15 | 87 | 100 | 56 |  |
| 14 d 16 | 86 | 64 | 72 |  |
| IF group | | | | |
| Baseline (n=16) | | | | |
| Baseline 1 | 5 | 6 | 8 |  |
| Baseline 2 | 6 | 5 | 7 |  |
| Baseline 3 | 7 | 4 | 7 |  |
| Baseline 4 | 8 | 7 | 7 |  |
| Baseline 5 | 5 | 3 | 5 |  |
| Baseline 6 | 4 | 4 | 4 |  |
| Baseline 7 | 7 | 8 | 10 |  |
| Baseline 8 | 6 | 9 | 9 |  |
| Baseline 9 | 10 | 8 | 10 |  |
| Baseline 10 | 9 | 6 | 7 |  |
| Baseline 11 | 7 | 5 | 6 |  |
| Baseline 12 | 8 | 4 | 5 |  |
| Baseline 13 | 5 | 3 | 4 |  |
| Baseline 14 | 3 | 8 | 3 |  |
| Baseline 15 | 2 | 7 | 6 |  |
| Baseline 16 | 2 | 4 | 4 |  |
| 24 h (n=16) | | | | |
| 24 h 1 | 120 | 108 | 98 |  |
| 24 h 2 | 87 | 120 | 120 |  |
| 24 h 3 | 112 | 80 | 106 |  |
| 24 h 4 | 109 | 78 | 97 |  |
| 24 h 5 | 83 | 95 | 86 |  |
| 24 h 6 | 75 | 56 | 68 |  |
| 24 h 7 | 120 | 120 | 120 |  |
| 24 h 8 | 86 | 115 | 98 |  |
| 24 h 9 | 116 | 83 | 109 |  |
| 24 h 10 | 115 | 100 | 109 |  |
| 24 h 11 | 89 | 92 | 84 |  |
| 24 h 12 | 89 | 120 | 95 |  |
| 24 h 13 | 86 | 115 | 112 |  |
| 24 h 14 | 56 | 98 | 108 |  |
| 24 h 15 | 103 | 73 | 80 |  |
| 24 h 16 | 90 | 93 | 88 |  |
| 7 d (n=16) | | | | |
| 7 d 1 | 65 | 65 | 76 |  |
| 7 d 2 | 80 | 74 | 60 |  |
| 7 d 3 | 68 | 89 | 65 |  |
| 7 d 4 | 82 | 62 | 42 |  |
| 7 d 5 | 63 | 43 | 57 |  |
| 7 d 6 | 35 | 56 | 54 |  |
| 7 d 7 | 105 | 98 | 118 |  |
| 7 d 8 | 56 | 97 | 108 |  |
| 7 d 9 | 47 | 76 | 58 |  |
| 7 d 10 | 82 | 76 | 23 |  |
| 7 d 11 | 15 | 65 | 57 |  |
| 7 d 12 | 75 | 46 | 73 |  |
| 7 d 13 | 75 | 64 | 38 |  |
| 7 d 14 | 74 | 27 | 68 |  |
| 7 d 15 | 65 | 76 | 87 |  |
| 7 d 16 | 63 | 75 | 22 |  |
| 14 d (n=16) | | | | |
| 14 d 1 | 46 | 72 | 55 |  |
| 14 d 2 | 30 | 54 | 30 |  |
| 14 d 3 | 62 | 67 | 72 |  |
| 14 d 4 | 79 | 24 | 28 |  |
| 14 d 5 | 43 | 32 | 36 |  |
| 14 d 6 | 43 | 54 | 37 |  |
| 14 d 7 | 57 | 25 | 78 |  |
| 14 d 8 | 64 | 87 | 98 |  |
| 14 d 9 | 47 | 86 | 75 |  |
| 14 d 10 | 71 | 67 | 56 |  |
| 14 d 11 | 45 | 58 | 85 |  |
| 14 d 12 | 64 | 75 | 57 |  |
| 14 d 13 | 65 | 72 | 47 |  |
| 14 d 14 | 54 | 38 | 76 |  |
| 14 d 15 | 56 | 71 | 43 |  |
| 14 d 16 | 34 | 75 | 28 |  |
| IF + SB group | | | | |
| Baseline (n=16) | | | | |
| Baseline 1 | 6 | 8 | 8 |  |
| Baseline 2 | 7 | 7 | 8 |  |
| Baseline 3 | 8 | 6 | 5 |  |
| Baseline 4 | 5 | 7 | 7 |  |
| Baseline 5 | 4 | 4 | 4 |  |
| Baseline 6 | 6 | 6 | 7 |  |
| Baseline 7 | 9 | 6 | 8 |  |
| Baseline 8 | 7 | 10 | 7 |  |
| Baseline 9 | 6 | 7 | 6 |  |
| Baseline 10 | 7 | 9 | 3 |  |
| Baseline 11 | 5 | 10 | 2 |  |
| Baseline 12 | 4 | 3 | 1 |  |
| Baseline 13 | 7 | 4 | 5 |  |
| Baseline 14 | 5 | 10 | 6 |  |
| Baseline 15 | 8 | 7 | 7 |  |
| Baseline 16 | 3 | 6 | 10 |  |
| 24 h (n=16) | | | | |
| 24 h 1 | 109 | 120 | 99 |  |
| 24 h 2 | 88 | 112 | 112 |  |
| 24 h 3 | 109 | 120 | 109 |  |
| 24 h 4 | 103 | 120 | 120 |  |
| 24 h 5 | 119 | 120 | 120 |  |
| 24 h 6 | 73 | 98 | 98 |  |
| 24 h 7 | 109 | 120 | 118 |  |
| 24 h 8 | 120 | 120 | 120 |  |
| 24 h 9 | 109 | 120 | 120 |  |
| 24 h 10 | 109 | 109 | 112 |  |
| 24 h 11 | 81 | 98 | 76 |  |
| 24 h 12 | 120 | 120 | 100 |  |
| 24 h 13 | 76 | 115 | 115 |  |
| 24 h 14 | 120 | 120 | 97 |  |
| 24 h 15 | 46 | 80 | 68 |  |
| 24 h 16 | 98 | 115 | 120 |  |
| 7 d (n=16) | | | | |
| 7 d 1 | 68 | 103 | 76 |  |
| 7 d 2 | 85 | 79 | 67 |  |
| 7 d 3 | 119 | 104 | 68 |  |
| 7 d 4 | 120 | 112 | 85 |  |
| 7 d 5 | 63 | 50 | 64 |  |
| 7 d 6 | 65 | 86 | 76 |  |
| 7 d 7 | 115 | 108 | 120 |  |
| 7 d 8 | 56 | 89 | 76 |  |
| 7 d 9 | 76 | 47 | 98 |  |
| 7 d 10 | 120 | 115 | 120 |  |
| 7 d 11 | 92 | 89 | 120 |  |
| 7 d 12 | 97 | 120 | 85 |  |
| 7 d 13 | 88 | 57 | 89 |  |
| 7 d 14 | 78 | 67 | 93 |  |
| 7 d 15 | 58 | 87 | 68 |  |
| 7 d 16 | 90 | 98 | 56 |  |
| 14 d (n=16) | | | | |
| 14 d 1 | 78 | 95 | 76 |  |
| 14 d 2 | 78 | 76 | 57 |  |
| 14 d 3 | 108 | 78 | 98 |  |
| 14 d 4 | 98 | 120 | 84 |  |
| 14 d 5 | 43 | 89 | 47 |  |
| 14 d 6 | 56 | 76 | 62 |  |
| 14 d 7 | 81 | 97 | 85 |  |
| 14 d 8 | 72 | 75 | 45 |  |
| 14 d 9 | 76 | 87 | 18 |  |
| 14 d 10 | 102 | 98 | 117 |  |
| 14 d 11 | 87 | 121 | 88 |  |
| 14 d 12 | 97 | 60 | 85 |  |
| 14 d 13 | 76 | 98 | 54 |  |
| 14 d 14 | 65 | 87 | 38 |  |
| 14 d 15 | 75 | 73 | 51 |  |
| 14 d 16 | 87 | 91 | 63 |  |
|  |  |  |  |  |

|  |  |  |  |  |
| --- | --- | --- | --- | --- |
| SMAD23/pSMAD23 Western blot | | | | |
| AL group (n=8) | | | | |
|  | SMAD23 | pSMAD23 | GAPDH |  |
| AL 1 | 33746.78 | 55678.51 | 63697.97 |  |
| AL 2 | 34521.21 | 58364.87 | 58766.02 |  |
| AL 3 | 35776.89 | 59387.25 | 62832.38 |  |
| AL 4 | 37466.74 | 56354.2 | 65647.88 |  |
| AL 5 | 38762.83 | 52873.85 | 64273.12 |  |
| AL 6 | 31994.56 | 51763.77 | 60985.65 |  |
| AL 7 | 30989.67 | 51287.19 | 64873.62 |  |
| AL 8 | 30892.01 | 52871.63 | 61364.26 |  |
| IF group (n=8) | | | | |
| IF 1 | 72368.58 | 88725.28 | 66390.78 |  |
| IF 2 | 76463.31 | 90827.83 | 65387.93 |  |
| IF 3 | 79475.52 | 95738.92 | 63872.37 |  |
| IF 4 | 80984.63 | 100927.31 | 62783.81 |  |
| IF 5 | 67463.26 | 84873.82 | 68372.98 |  |
| IF 6 | 50465.13 | 86374.75 | 67008.74 |  |
| IF 7 | 69847.8 | 81829.34 | 61928.22 |  |
| IF 8 | 68336.44 | 80923.82 | 60879.16 |  |
| IF+SB group (n=8) | | | | |
| IF+SB 1 | 56621.95 | 47437.04 | 60572.88 |  |
| IF+SB 2 | 68736.83 | 58948.97 | 59837.72 |  |
| IF+SB 3 | 50808.02 | 54733.88 | 58108.33 |  |
| IF+SB 4 | 72893.27 | 57362.15 | 62763.87 |  |
| IF+SB 5 | 69334.52 | 50984.83 | 63273.28 |  |
| IF+SB 6 | 53748.84 | 40837.73 | 66876.98 |  |
| IF+SB 7 | 60984.63 | 41886.54 | 65352.91 |  |
| IF+SB 8 | 51293.46 | 42531.13 | 67263.76 |  |
|  |  |  |  |  |

|  |  |  |  |  |  |  |  |  |  |  |
| --- | --- | --- | --- | --- | --- | --- | --- | --- | --- | --- |
| CD31/Ki67 Immunofluorescence (cells/field) | | | | | | | | | | |
| AL group (n=8) | | | | | | | | | | |
| AL 1 | 4 | 5 | 3 | 6 | 8 | 4 | 2 | 0 | 2 | 6 |
| AL 2 | 4 | 6 | 8 | 4 | 2 | 4 | 1 | 9 | 4 | 6 |
| AL 3 | 4 | 5 | 7 | 8 | 10 | 5 | 4 | 9 | 9 | 5 |
| AL 4 | 3 | 4 | 5 | 6 | 3 | 4 | 3 | 4 | 5 | 9 |
| AL 5 | 2 | 4 | 1 | 1 | 7 | 6 | 4 | 6 | 6 | 6 |
| AL 6 | 7 | 8 | 6 | 5 | 8 | 2 | 11 | 3 | 5 | 10 |
| AL 7 | 5 | 8 | 7 | 1 | 5 | 2 | 3 | 2 | 4 | 5 |
| AL 8 | 8 | 4 | 5 | 6 | 7 | 3 | 5 | 6 | 6 | 8 |
| IF group (n=8) | | | | | | | | | | |
| IF 1 | 6 | 8 | 9 | 0 | 8 | 6 | 2 | 5 | 7 | 5 |
| IF 2 | 7 | 9 | 8 | 4 | 10 | 11 | 12 | 9 | 10 | 12 |
| IF 3 | 3 | 7 | 9 | 8 | 11 | 13 | 14 | 12 | 10 | 11 |
| IF 4 | 5 | 6 | 10 | 13 | 8 | 14 | 6 | 8 | 16 | 8 |
| IF 5 | 7 | 9 | 8 | 8 | 9 | 6 | 11 | 5 | 12 | 11 |
| IF 6 | 5 | 4 | 3 | 6 | 4 | 6 | 2 | 8 | 1 | 1 |
| IF 7 | 11 | 13 | 13 | 9 | 4 | 6 | 12 | 8 | 9 | 15 |
| IF 8 | 11 | 0 | 9 | 8 | 6 | 5 | 2 | 9 | 8 | 10 |
| IF+SB group (n=8) | | | | | | | | | | |
| IF+SB 1 | 1 | 7 | 0 | 2 | 2 | 0 | 6 | 6 | 4 | 5 |
| IF+SB 2 | 0 | 4 | 0 | 2 | 8 | 0 | 4 | 7 | 3 | 4 |
| IF+SB 3 | 5 | 6 | 6 | 8 | 3 | 2 | 3 | 3 | 6 | 3 |
| IF+SB 4 | 4 | 0 | 5 | 7 | 1 | 4 | 2 | 3 | 4 | 6 |
| IF+SB 5 | 9 | 7 | 1 | 5 | 2 | 3 | 3 | 2 | 2 | 2 |
| IF+SB 6 | 3 | 2 | 1 | 0 | 3 | 8 | 4 | 6 | 1 | 1 |
| IF+SB 7 | 5 | 6 | 2 | 6 | 6 | 3 | 1 | 2 | 3 | 2 |
| IF+SB 8 | 2 | 1 | 6 | 1 | 7 | 0 | 1 | 1 | 6 | 5 |
|  |  |  |  |  |  |  |  |  |  |  |

|  |  |  |  |  |
| --- | --- | --- | --- | --- |
| r CBF (%) | | | | |
| AL group (n=8) | | | | |
|  | Baseline | 24 h | 7 d | 14 d |
| AL 1 | 100 | 24 | 27 | 38 |
| AL 2 | 100 | 25 | 31 | 53 |
| AL 3 | 100 | 28 | 33 | 59 |
| AL 4 | 100 | 27 | 64 | 60 |
| AL 5 | 100 | 21 | 35 | 34 |
| AL 6 | 100 | 20 | 35 | 47 |
| AL 7 | 100 | 27 | 54 | 36 |
| AL 8 | 100 | 27 | 42 | 43 |
| IF group (n=8) | | | | |
| IF 1 | 100 | 26 | 46 | 65 |
| IF 2 | 100 | 27 | 57 | 80 |
| IF 3 | 100 | 21 | 75 | 75 |
| IF 4 | 100 | 21 | 76 | 63 |
| IF 5 | 100 | 25 | 36 | 60 |
| IF 6 | 100 | 28 | 54 | 39 |
| IF 7 | 100 | 29 | 55 | 54 |
| IF 8 | 100 | 28 | 53 | 73 |
| IF+SB group (n=8) | | | | |
| IF+SB 1 | 100 | 21 | 26 | 56 |
| IF+SB 2 | 100 | 26 | 30 | 34 |
| IF+SB 3 | 100 | 25 | 35 | 36 |
| IF+SB 4 | 100 | 25 | 45 | 32 |
| IF+SB 5 | 100 | 27 | 55 | 37 |
| IF+SB 6 | 100 | 22 | 37 | 40 |
| IF+SB 7 | 100 | 21 | 30 | 51 |
| IF+SB 8 | 100 | 29 | 45 | 30 |
|  |  |  |  |  |

|  |  |  |  |  |  |  |  |  |  |  |
| --- | --- | --- | --- | --- | --- | --- | --- | --- | --- | --- |
| Total vessel surface area | | | | | | | | | | |
| AL group (n=8) | | | | | | | | | | |
| AL 1 | 0.58 | 0.52 | 0.53 | 0.56 | 0.5 | 0.6 | 0.54 | 0.47 |  |  |
| AL 2 | 0.51 | 0.59 | 0.38 | 0.48 | 0.5 | 0.48 | 0.32 | 0.39 |  |  |
| AL 3 | 0.49 | 0.39 | 0.48 | 0.5 | 0.35 | 0.44 | 0.48 | 0.43 |  |  |
| AL 4 | 0.38 | 0.48 | 0.42 | 0.51 | 0.46 | 0.48 | 0.46 | 0.53 |  |  |
| AL 5 | 0.65 | 0.71 | 0.48 | 0.48 | 0.5 | 0.45 | 0.43 | 0.55 |  |  |
| AL 6 | 0.58 | 0.46 | 0.37 | 0.39 | 0.4 | 0.42 | 0.44 | 0.47 |  |  |
| AL 7 | 0.87 | 0.68 | 0.51 | 0.42 | 0.43 | 0.46 | 0.48 | 0.5 |  |  |
| AL 8 | 0.49 | 0.43 | 0.38 | 0.32 | 0.38 | 0.35 | 0.39 | 0.44 |  |  |
| IF group (n=8) | | | | | | | | | | |
| IF 1 | 0.63 | 0.64 | 0.53 | 0.59 | 0.6 | 0.6 | 0.61 | 0.67 |  |  |
| IF 2 | 0.52 | 0.6 | 0.47 | 0.59 | 0.6 | 0.61 | 0.65 | 0.46 |  |  |
| IF 3 | 0.58 | 0.49 | 0.67 | 0.57 | 0.7 | 0.68 | 0.69 | 0.5 |  |  |
| IF 4 | 0.53 | 0.54 | 0.58 | 0.52 | 0.48 | 0.49 | 0.55 | 0.52 |  |  |
| IF 5 | 0.65 | 0.71 | 0.58 | 0.57 | 0.65 | 0.64 | 0.63 | 0.62 |  |  |
| IF 6 | 0.82 | 0.28 | 0.8 | 0.73 | 0.73 | 0.79 | 0.61 | 0.52 |  |  |
| IF 7 | 0.38 | 0.49 | 0.76 | 0.54 | 0.66 | 0.62 | 0.78 | 0.54 |  |  |
| IF 8 | 0.6 | 0.63 | 0.63 | 0.68 | 0.7 | 0.61 | 0.54 | 0.59 |  |  |
| IF+SB group (n=8) | | | | | | | | | | |
| IF+SB 1 | 0.48 | 0.48 | 0.5 | 0.51 | 0.41 | 0.45 | 0.38 | 0.46 |  |  |
| IF+SB 2 | 0.43 | 0.41 | 0.32 | 0.49 | 0.47 | 0.55 | 0.51 | 0.52 |  |  |
| IF+SB 3 | 0.39 | 0.37 | 0.31 | 0.34 | 0.33 | 0.4 | 0.49 | 0.38 |  |  |
| IF+SB 4 | 0.39 | 0.38 | 0.38 | 0.41 | 0.48 | 0.48 | 0.53 | 0.56 |  |  |
| IF+SB 5 | 0.48 | 0.47 | 0.43 | 0.17 | 0.25 | 0.38 | 0.48 | 0.5 |  |  |
| IF+SB 6 | 0.62 | 0.15 | 0.39 | 0.24 | 0.42 | 0.25 | 0.29 | 0.31 |  |  |
| IF+SB 7 | 0.64 | 0.26 | 0.18 | 0.46 | 0.35 | 0.26 | 0.19 | 0.23 |  |  |
| IF+SB 8 | 0.4 | 0.48 | 0.48 | 0.5 | 0.38 | 0.46 | 0.48 | 0.49 |  |  |
|  |  |  |  |  |  |  |  |  |  |  |

|  |  |  |  |  |  |  |  |  |  |  |
| --- | --- | --- | --- | --- | --- | --- | --- | --- | --- | --- |
| Number of branch points | | | | | | | | | | |
| AL group (n=8) | | | | | | | | | | |
| AL 1 | 67 | 74 | 45 | 76 | 57 | 76 | 64 | 66 |  |  |
| AL 2 | 56 | 44 | 65 | 54 | 48 | 47 | 43 | 49 |  |  |
| AL 3 | 66 | 60 | 65 | 63 | 57 | 55 | 59 | 54 |  |  |
| AL 4 | 62 | 68 | 62 | 41 | 54 | 58 | 57 | 55 |  |  |
| AL 5 | 60 | 64 | 81 | 68 | 78 | 66 | 76 | 83 |  |  |
| AL 6 | 90 | 81 | 56 | 43 | 48 | 47 | 73 | 55 |  |  |
| AL 7 | 68 | 78 | 73 | 69 | 81 | 83 | 58 | 66 |  |  |
| AL 8 | 68 | 69 | 67 | 64 | 61 | 54 | 58 | 56 |  |  |
| IF group (n=8) | | | | | | | | | | |
| IF 1 | 87 | 88 | 83 | 71 | 94 | 76 | 79 | 88 |  |  |
| IF 2 | 78 | 60 | 85 | 68 | 87 | 61 | 81 | 83 |  |  |
| IF 3 | 78 | 74 | 67 | 72 | 83 | 69 | 86 | 82 |  |  |
| IF 4 | 68 | 87 | 81 | 90 | 83 | 56 | 89 | 92 |  |  |
| IF 5 | 82 | 76 | 47 | 78 | 86 | 49 | 78 | 69 |  |  |
| IF 6 | 93 | 102 | 67 | 65 | 89 | 59 | 96 | 80 |  |  |
| IF 7 | 98 | 92 | 105 | 84 | 79 | 97 | 81 | 88 |  |  |
| IF 8 | 85 | 75 | 98 | 87 | 82 | 84 | 76 | 65 |  |  |
| IF+SB group (n=8) | | | | | | | | | | |
| IF+SB 1 | 54 | 28 | 71 | 65 | 76 | 65 | 66 | 74 |  |  |
| IF+SB 2 | 67 | 17 | 65 | 75 | 46 | 62 | 59 | 51 |  |  |
| IF+SB 3 | 52 | 47 | 57 | 49 | 68 | 20 | 58 | 51 |  |  |
| IF+SB 4 | 45 | 65 | 57 | 67 | 32 | 54 | 46 | 49 |  |  |
| IF+SB 5 | 67 | 55 | 14 | 29 | 59 | 52 | 48 | 63 |  |  |
| IF+SB 6 | 78 | 36 | 39 | 53 | 64 | 72 | 47 | 42 |  |  |
| IF+SB 7 | 27 | 71 | 16 | 35 | 30 | 42 | 47 | 38 |  |  |
| IF+SB 8 | 65 | 54 | 51 | 42 | 16 | 48 | 44 | 51 |  |  |
|  |  |  |  |  |  |  |  |  |  |  |
