# Supplementary figures and images for: Long-term intermittent fasting improves neurological function by promoting angiogenesis after cerebral ischemia via growth differentiation factor 11 signaling activation
Source: PLoS One. 2023 Mar 30;18(3):e0282338. doi: 10.1371/journal.pone.0282338 (PMC10062670; doi:10.1371/journal.pone.0282338)

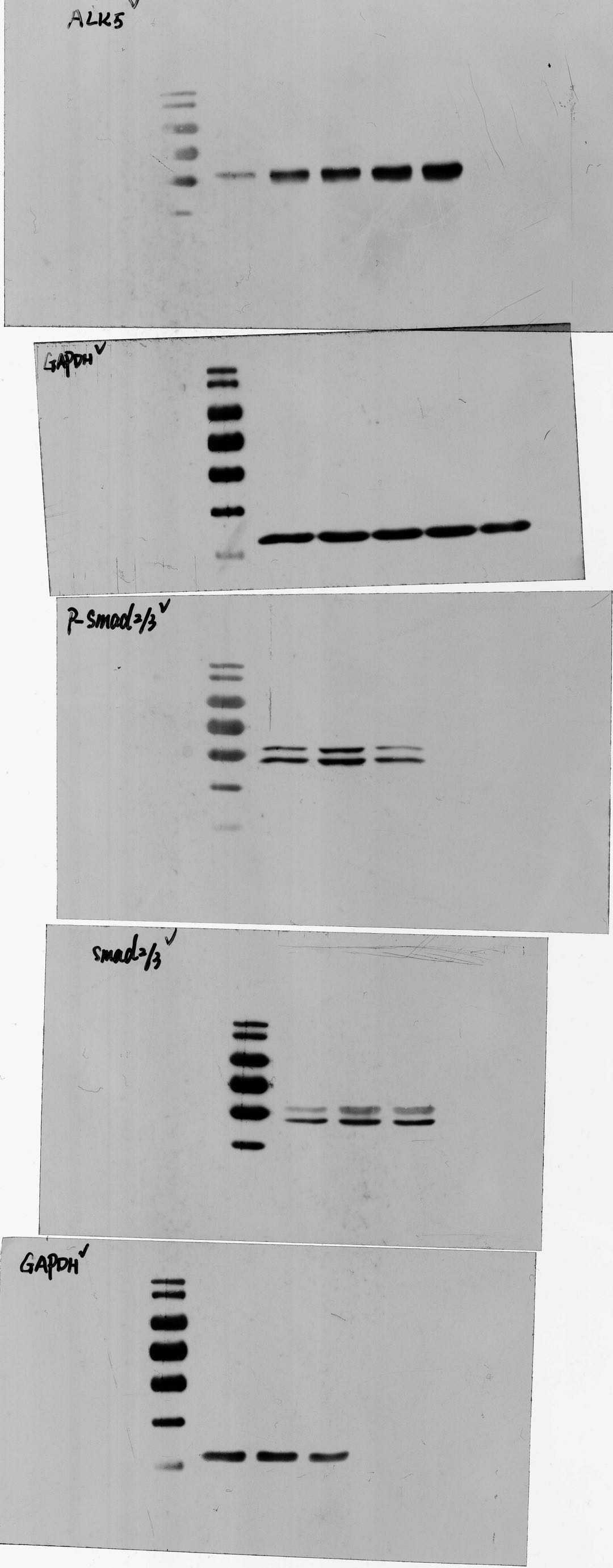

Supplement: S1 Raw image — (JPG) [file pone.0282338.s003.jpg]
